# Precision immuno-oncology in triple-negative breast cancer: from PD-L1 to multimodal biomarkers, ADC–ICI combinations, and global real-world integration

**DOI:** 10.3389/fimmu.2026.1911879

**Published:** 2026-10-12

**Authors:** Jingna Wu

**Affiliations:** Department of Breast Oncology, Center for Cancer Prevention and Treatment, Meizhou People’s Hospital (Huangtang Hospital), Meizhou Academy of Medical Sciences, Meizhou, China

**Keywords:** antibody-drug conjugates, biomarkers, health equity, immune checkpoint inhibitors, immunotherapy, liquid biopsy, real-world evidence, resistance

## Abstract

Immune checkpoint blockade improves outcomes in defined settings of triple-negative breast cancer (TNBC), but treatment failure does not identify a single biological mechanism or an interchangeable set of remedies. This focused narrative review examines how biomarkers can distinguish established treatment eligibility from investigational strategies intended to address immune exclusion and resistance. Randomized TNBC trials provide the clinical reference, while translational studies and trials in other malignancies are classified according to their applicability. PD-L1 testing remains relevant to first-line pembrolizumab combinations in advanced TNBC; it is not required to select patients for the KEYNOTE-522 early-stage regimen. Sacituzumab govitecan plus pembrolizumab is a United States-approved first-line option for PD-L1-positive advanced disease, but its comparative benefit establishes regimen-level efficacy rather than immunological synergy or reversal of checkpoint resistance. Proposed immune-priming effects remain specific to the ADC, experimental model and disease setting and should not be generalized across the ADC class. Stromal lymphocytes, spatial immune organization and circulating tumor DNA provide complementary biological or prognostic information without establishing a general replacement for PD-L1 or a validated rule for treatment escalation or omission. Strategies involving epigenetic modulation, adenosine blockade, DNA-damage signaling and local immune priming show different levels of evidence, including negative randomized findings. Particular caution is needed when extrapolating antigen-density thresholds and cellular-therapy outcomes from hematological malignancies. A useful biomarker framework must specify the disease setting, assay, proposed action and evidence that changing treatment improves outcomes. Prospective testing of these links, together with regimen-specific safety assessment and reliable access to established diagnostics, is necessary before resistance-directed combinations can be implemented routinely.

## 1 Introduction

Immune checkpoint inhibitors (ICIs) have changed systemic treatment for selected patients with triple-negative breast cancer (TNBC). KEYNOTE-522 established a perioperative pembrolizumab regimen in high-risk early disease, and KEYNOTE-355 established a survival benefit for pembrolizumab plus chemotherapy in the PD-L1 combined positive score (CPS) ≥10 population with advanced disease. These results support different decisions in different clinical settings; they do not establish a common biomarker threshold across all stages of TNBC (1, 2).

The central problem considered here is how to identify and address inadequate antitumor immune activity without promoting unvalidated biomarkers or combinations into routine care. A tumor may lack effective immune infiltration, contain lymphocytes that are spatially excluded from cancer cells, or progress after an initially effective treatment. These states suggest different research questions. They cannot be distinguished by simply describing all nonresponders as having a “cold” tumor, nor can an immune-rich biopsy establish that checkpoint blockade will add benefit. Spatial analyses in TNBC provide a basis for examining these distinctions, but translating a biological association into a treatment decision requires additional evidence (3).

We therefore focus on biomarker-guided strategies to address immune exclusion and resistance. ADC combinations are discussed as potential means of coupling tumor killing with immune activation; established trials define the clinical benchmark against which these strategies should be judged. Real-world delivery and toxicity are considered where they affect the feasibility of a biomarker-directed decision. Cellular therapies, vaccines and digital monitoring are examined where they clarify antigen recognition, evidence requirements or the delivery of care. The contribution of this review is a structured assessment of the links between a measured abnormality, a proposed intervention and the evidence needed to justify that intervention.

### 1.1 Biological heterogeneity and the meaning of precision

TNBC is a receptor-defined diagnosis rather than a single immune state. A tumor can satisfy the pathological definition while differing substantially from another TNBC in malignant-cell differentiation, DNA repair, stromal composition and immune organization. This distinction matters because a drug may require a specific target or an intact immune process that is not specified by receptor negativity. The refinement of the Lehmann transcriptional classification illustrates the problem: signals originally interpreted as immunomodulatory and mesenchymal stem-like tumor subtypes were substantially contributed by infiltrating lymphocytes and stromal cells. Bulk expression therefore describes both the cancer and the sampled ecosystem. It cannot always identify which compartment should be treated (4).

For this review, precision means matching a reproducible observation to a defined clinical decision. The observation may establish eligibility, identify prognosis, document an on-treatment change or suggest a resistance mechanism. These functions should remain distinct. A baseline high-risk marker may identify a population with a large absolute opportunity for benefit without predicting a larger relative treatment effect. A pharmacodynamic marker may demonstrate that a drug reaches its biological target without demonstrating that the target matters for survival. A response-associated signature obtained after treatment may be unsuitable for pretreatment selection. Maintaining these distinctions permits a broad discussion of biomarkers, combination treatments and implementation while keeping a common analytical question throughout: what evidence would justify acting on this finding?

### 1.2 An immune ecosystem that changes during treatment

Multiplex imaging in TNBC demonstrated organized relationships between malignant and immune compartments rather than a random distribution of infiltrating cells. Single-cell analyses of breast tumors before and during PD-1 blockade subsequently revealed heterogeneous cellular changes, while a separate TNBC investigation associated particular immune-cell populations with response to PD-L1-containing treatment. Together, these studies support serial, compartment-aware analysis. They do not establish one universally favorable cell population across assays, disease stages and regimens. The same increase in lymphocyte abundance may represent useful recruitment, redistribution or expansion of cells that remain ineffective at recognizing the tumor (5–7).

This dynamic perspective also changes the interpretation of progression. A progressing lesion after chemoimmunotherapy might have lost susceptibility to the cytotoxic component, acquired a defect in immune recognition or simply received inadequate drug exposure. Different lesions may follow different trajectories. A research biopsy should therefore be linked to its anatomical site, treatment history and timing, and ideally compared with pretreatment tissue. The aim is not to require repeated invasive sampling in every patient, but to avoid assigning a biological mechanism from a clinical outcome alone.

## 2 Literature identification and evidence interpretation

For this revision, targeted electronic searches were undertaken on 8 September 2026 to identify PubMed-indexed publications, original journal reports, ClinicalTrials.gov records and official regulatory documents. Search concepts combined TNBC or triple-negative breast cancer with pembrolizumab, PD-L1 assay comparison, spatial predictors, tumor-infiltrating lymphocytes, circulating tumor DNA, immune resistance, antibody-drug conjugates, epigenetic priming and adenosine blockade. Named searches were also performed for KEYNOTE-522, KEYNOTE-355, ASCENT-03, ASCENT-04, SASCIA, BEGONIA, c-TRAK TN, TONIC, SYNERGY, IMpassion030, IMpassion031, GeparNuevo, NeoPACT, individualized vaccination studies and the two publications suggested during peer review. Additional searches addressed ADC resistance, longitudinal ctDNA studies, patient-reported monitoring and methodological reporting standards. Foundational studies were retained when needed to establish the origin of a mechanism; recent reports were prioritized for clinical results and regulatory status. These targeted searches were used to update and verify the evidence for the present narrative synthesis. For the present correction, targeted searches on 12 September 2026 additionally identified methodological literature on transcriptomic classifier prespecification, independent validation and bias from gene selection during cross-validation.

Selection prioritized randomized TNBC clinical trials, primary translational studies with identifiable patient populations, prospective biomarker studies, toxicity guidance and trial registrations that resolved questions about design. Non-TNBC studies were included only for explicitly identified mechanistic or safety context. Registry descriptions were used to establish what a trial tests, not to infer efficacy. Conference reports were distinguished from full publications. Claims with no adequate supporting source were removed or recast as questions for future research. The review is not a systematic review or meta-analysis: there was no exhaustive retrieval, duplicate independent screening, formal risk-of-bias scoring or quantitative pooling. Selective inclusion and publication bias therefore remain possible.

Evidence is discussed in four categories: randomized clinical evidence in TNBC; nonrandomized or exploratory clinical evidence in TNBC; preclinical evidence in TNBC or breast cancer models; and indirect evidence from other malignancies. These categories describe applicability and design, not a formal GRADE assessment. Each biomarker is evaluated separately for analytical validity, association with outcome, prediction of a differential treatment effect and demonstrated clinical utility. In particular, a marker associated with response among ICI-treated patients is not automatically predictive of the benefit added by the ICI. That inference requires an appropriate comparator and a treatment-by-biomarker analysis.

## 3 Clinical benchmarks for interpreting resistance directed strategies

### 3.1 Early disease and the limits of response guided omission

KEYNOTE-522 randomized 1,174 patients with previously untreated stage II or III TNBC. Its regimen combined pembrolizumab with paclitaxel and carboplatin, followed by anthracycline and cyclophosphamide, with pembrolizumab continued after surgery. In the initial pathological complete response analysis, rates were 64.8% and 51.2% with pembrolizumab and placebo, respectively. A subsequent overall survival report showed 60-month survival of 86.6% versus 81.7% and a hazard ratio for death of 0.66 (95% CI 0.50–0.87). Thus, the evidence is no longer limited to a trend in overall survival (1, 8).

The clinically important distinction is between benefit from the tested perioperative strategy and benefit from each component considered separately. KEYNOTE-522 did not rerandomize patients after surgery to establish whether adjuvant pembrolizumab could safely be omitted after a pathological complete response. Neither pathological complete response nor ctDNA negativity alone establishes absence of additional treatment benefit. In particular, no prospective interventional evidence supports omission of adjuvant pembrolizumab solely on the basis of a negative ctDNA result. Biomarker-guided omission requires prospective comparative evidence demonstrating preservation of clinically meaningful outcomes (8, 9, 51).

PD-L1 positivity is not an eligibility requirement for this early-stage regimen. Importing the metastatic CPS ≥10 threshold into this setting would exclude patients on a basis not used to define the established treatment population. Conversely, the early-stage evidence does not authorize ICI use across all metastatic PD-L1 categories (2, 8).

The exploratory residual cancer burden analysis of KEYNOTE-522 adds granularity to the binary pCR endpoint. Residual cancer burden incorporates the extent of residual breast and nodal disease, enabling assessment of event-free survival across postoperative categories. However, those categories are measured after randomized treatment and are themselves affected by treatment. Comparing outcomes within a residual-disease category does not recreate randomization or identify the independent effect of postoperative pembrolizumab. The analysis is informative for risk characterization and hypothesis generation, but should not be converted into a validated rule to omit or add a treatment for an individual category (10).

Other early-stage trials help test whether the benefit is a general property of any checkpoint inhibitor delivered at any time. IMpassion031 improved pCR with perioperative atezolizumab, and its later report added survival and ctDNA analyses; these secondary analyses should be interpreted within their stated statistical framework. GeparNuevo investigated durvalumab with neoadjuvant chemotherapy without the same prolonged postoperative checkpoint schedule. Its long-term analysis contributes to the question of treatment timing, but a phase II trial cannot establish equivalence to the KEYNOTE-522 regimen. Conversely, ALEXANDRA/IMpassion030 did not show benefit from adding atezolizumab to chemotherapy after surgery. Taken together, these trials caution against transferring a perioperative result to an adjuvant-only strategy. They also do not isolate timing from differences in agent, chemotherapy and population (11–13).

The de-escalation question should specify the component being reduced. NeoPACT investigated pembrolizumab with carboplatin and docetaxel as an anthracycline-free neoadjuvant strategy and included exploratory immune biomarker assessments. This is materially different from omitting all chemotherapy or discontinuing postoperative immunotherapy according to ctDNA. Its phase II findings support further comparison and provide information for patients in whom an anthracycline is problematic, but do not establish noninferiority to a randomized standard. A de-escalation trial must measure both the toxicity avoided and the disease-control risk accepted; a favorable response proportion alone is insufficient (14).

### 3.2 Advanced disease and assay specific eligibility

KEYNOTE-355 compared pembrolizumab or placebo with paclitaxel, nab-paclitaxel, or gemcitabine plus carboplatin. In the CPS ≥10 subgroup, median overall survival was 23.0 versus 16.1 months (hazard ratio 0.73; 95% CI 0.55–0.95). Paclitaxel and nab-paclitaxel were individual alternatives, while gemcitabine and carboplatin formed the doublet; these distinctions matter when interpreting the regimen comparison (2).

For pembrolizumab selection, CPS counts PD-L1-staining tumor cells, lymphocytes and macrophages relative to viable tumor cells, multiplied by 100. This differs from the SP142 immune-cell algorithm used in IMpassion130, which assesses the tumor area occupied by stained immune cells. These scoring systems identify different biological and clinical measurements. The original IMpassion130 trial evaluated atezolizumab with nab-paclitaxel in this assay-defined context (15). A comparison within IMpassion130 identified substantially different positive populations across assays, demonstrating why changing the antibody or scoring algorithm can change eligibility (16).

IMpassion131 did not reproduce a progression-free survival benefit when atezolizumab was combined with conventional paclitaxel. The finding cautions against assuming that checkpoint antibodies and chemotherapy partners are interchangeable. It does not constitute a study of pembrolizumab in an off-label PD-L1-negative real-world population. Atezolizumab’s United States TNBC indication was withdrawn; historical trial results must therefore be separated from current local prescribing permissions (17, 18).

The development of metastatic immunotherapy also illustrates why early activity signals require comparative confirmation. Atezolizumab monotherapy produced durable responses in a subset of patients in a phase I study, whereas KEYNOTE-119 did not establish an overall survival advantage for pembrolizumab over chemotherapy in its prespecified second- or third-line comparisons. A response in a selected early-phase cohort therefore cannot justify routine later-line checkpoint monotherapy. Treatment line, disease burden, eligibility and prior immune exposure must accompany any description of activity (19, 20).

KEYNOTE-355’s initial progression-free survival report and subsequent overall survival analysis represent complementary evidence from the same randomized population, not independent replications. Its hierarchical assessment of PD-L1 populations also means that evidence cannot be summarized merely as benefit in “PD-L1-positive” disease without the scoring system and threshold. The clinically useful statement identifies CPS, the relevant cutoff, the first-line setting and the tested chemotherapy partners. This level of specificity prevents a biomarker discussion from inadvertently widening an indication (2, 21).

### 3.3 The ADC benchmark has changed

ASCENT-04/KEYNOTE-D19 randomized 443 patients with previously untreated PD-L1-positive advanced TNBC to sacituzumab govitecan (SG) plus pembrolizumab or chemotherapy plus pembrolizumab. Median progression-free survival was 11.2 versus 7.8 months (hazard ratio 0.65; 95% CI 0.51–0.84). Overall survival was immature in the primary report. On 24 June 2026, the FDA approved the combination for first-line unresectable locally advanced or metastatic TNBC with CPS ≥10. This is a United States regulatory statement, not a claim of universal approval or reimbursement (22, 23).

ASCENT-04/KEYNOTE-D19 establishes the comparative efficacy of SG plus pembrolizumab versus chemotherapy plus pembrolizumab in the trial-defined population. Because both groups received pembrolizumab and there was no SG-alone arm, the trial cannot estimate the benefit added by pembrolizumab to SG or distinguish differences in cytotoxic activity from an immune-mediated interaction. Its positive result therefore does not, by itself, demonstrate immunological synergy. Nor does it establish efficacy in patients progressing on prior PD-1/PD-L1 blockade or validate ICI rechallenge after perioperative pembrolizumab; these questions require evidence in explicitly defined ICI-exposed populations (22).

ASCENT-03 addressed a different question: SG monotherapy versus chemotherapy in patients not eligible for PD-1/PD-L1 inhibitor-based treatment. It provides a monotherapy benchmark for an ICI-ineligible population. SASCIA evaluates SG versus treatment of physician’s choice after neoadjuvant treatment in high-risk HER2-negative breast cancer with residual disease. Its postoperative design addresses recurrence risk rather than neoadjuvant pathological response. **Supplementary Table 1** separates these designs from combination trials (24, 25).

## 4 ADC–ICI combinations: agent-specific immune hypotheses and evidential limits

### 4.1 From tumor killing to immune activation

An ADC can link antigen-directed delivery to cytotoxic payload exposure. In a HER2-expressing mouse model, trastuzumab deruxtecan (T-DXd) increased markers associated with dendritic-cell activation, tumor antigen presentation and adaptive antitumor immunity. This supports a combination rationale but does not establish that every ADC produces the same immune effects in human TNBC. The relevant variables include target distribution, internalization, linker behavior, payload sensitivity and the immune competence of the experimental model (26).

Several mechanistic distinctions prevent overinterpretation. Calreticulin exposure at the cell surface should be distinguished from extracellular ATP or HMGB1 release. Dendritic cells cross-present antigen through peptide–MHC complexes that are recognized by T-cell receptors. Bystander killing describes payload activity in neighboring cells, whereas antigen spreading describes expansion of an immune response to additional antigens. Neither process proves the other. **Figure 1** therefore separates direct cytotoxicity from the hypothesized immune contribution. Regulatory T-cell depletion and macrophage reprogramming should likewise be interpreted as model-dependent observations rather than shared class effects (27).

**Figure 1:**
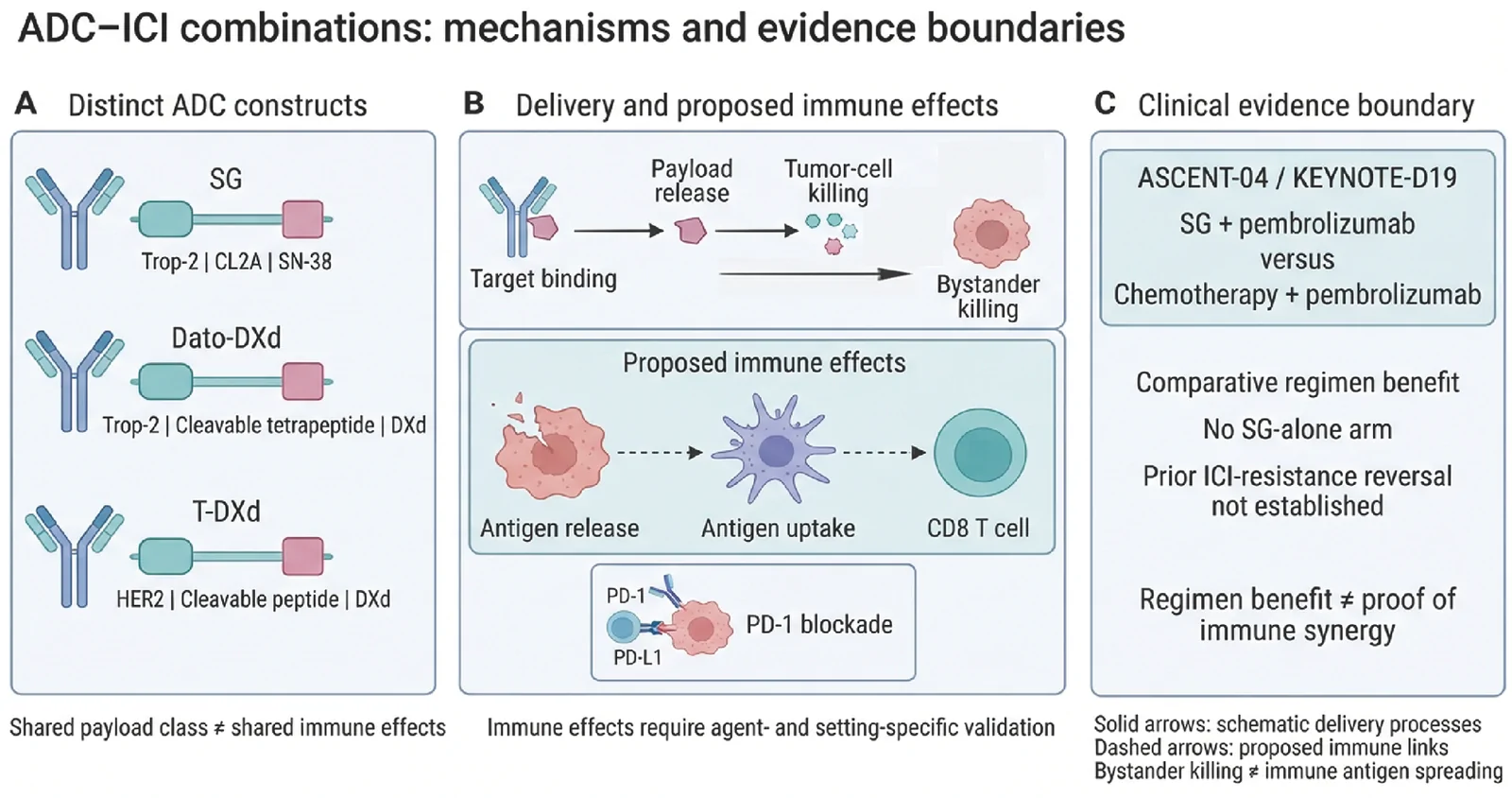
Agent-specific hypotheses and evidence boundaries for ADC–ICI combinations. **(A)** Sacituzumab govitecan (SG), datopotamab deruxtecan (Dato-DXd) and trastuzumab deruxtecan (T-DXd) differ in target antigen and/or linker–payload characteristics. Shared topoisomerase-I inhibition does not establish equivalent cytotoxic, bystander or immune effects. The constructs are schematic and do not represent chemical structures or drug-to-antibody ratios. **(B)** Target-directed payload delivery can produce tumor-cell killing and, depending on the agent and context, killing of neighboring cells. The dashed pathway illustrates proposed links between tumor injury, antigen uptake by dendritic cells and T-cell activation. These immune effects require confirmation for each ADC, experimental model and TNBC setting and should not be interpreted as universal properties of the ADC class. PD-1 blockade is shown as a separate combination component; its inclusion does not establish an immune-mediated interaction with the ADC. Bystander killing is distinct from immune antigen spreading. **(C)** ASCENT-04/KEYNOTE-D19 compares SG plus pembrolizumab with chemotherapy plus pembrolizumab. Its comparative benefit establishes regimen-level efficacy in the trial-defined population. Because both groups received pembrolizumab and there was no SG-alone arm, the trial does not isolate the benefit added by pembrolizumab to SG, prove immunological synergy or establish reversal of resistance to prior ICI therapy. Solid arrows indicate schematic cytotoxic delivery processes; dashed arrows indicate proposed immune links (22, 26–29). ADC, antibody–drug conjugate; ICI, immune checkpoint inhibitor; TNBC, triple-negative breast cancer; SG, sacituzumab govitecan; Dato-DXd, datopotamab deruxtecan; T-DXd, trastuzumab deruxtecan; HER2, human epidermal growth factor receptor 2; Trop-2, trophoblast cell-surface antigen 2; PD-1, programmed cell death protein 1.

For a resistance-directed trial, response alone is insufficient evidence that immune exclusion has been reversed. Paired tissue sampling could assess whether the combination changes intratumoral CD8 localization, antigen presentation or T-cell activation; these changes should then be related to outcomes and compared with an appropriate treatment arm. Otherwise, tumor shrinkage caused by the ADC could be mistakenly attributed to restored checkpoint sensitivity. This is a proposed experimental requirement rather than an established clinical test.

SG, Dato-DXd and T-DXd should not be treated as mechanistically interchangeable. SG targets Trop-2 and carries SN-38 through a hydrolysable CL2A linker, whereas Dato-DXd also targets Trop-2 but delivers the distinct topoisomerase-I inhibitor DXd through a protease-cleavable tetrapeptide linker. T-DXd uses a HER2-directed antibody with a cleavable peptide linker and DXd payload, making its targeting context different from that of the Trop-2-directed agents. Differences in antigen distribution, antibody properties, linker cleavage and payload exposure can alter both direct killing and bystander effects; their magnitude should not be assumed equivalent across agents or experimental systems. Likewise, the dendritic-cell and adaptive immune effects reported for T-DXd in a HER2-expressing mouse model cannot be assigned to SG or Dato-DXd without agent-specific evidence. Shared topoisomerase-I inhibition is insufficient to establish a shared immune-priming phenotype in human TNBC (26, 28, 29).

### 4.2 Separating agents and clinical settings

The phase III ASCENT trial established SG activity in previously treated metastatic TNBC, whereas ASCENT-04 tested its earlier use with pembrolizumab. Their populations and comparators address different treatment decisions. A favorable result for one regimen cannot be used as the efficacy reference for another ADC-ICI combination (22, 103).

For datopotamab deruxtecan (Dato-DXd), BEGONIA arms 7 and 8 have reported first-line combination results with durvalumab. These phase Ib/II data support further clinical development, but their response rates should not be ranked directly against randomized trial results with different eligibility criteria, PD-L1 selection and follow-up. TROPION-Breast05 is the specific randomized phase III design evaluating Dato-DXd with or without durvalumab against chemotherapy plus pembrolizumab in PD-L1-high advanced TNBC, with the monotherapy arm available in selected countries. A protocol publication establishes this comparison, not its outcome (30, 104).

T-DXd monotherapy has randomized evidence in previously treated HER2-low advanced breast cancer from DESTINY-Breast04, a study composed predominantly of hormone-receptor-positive disease with a smaller hormone-receptor-negative subgroup. Its results do not establish T-DXd-pembrolizumab efficacy in TNBC (31). The latter combination is registered under NCT04042701, providing a design reference rather than a TNBC-specific efficacy estimate (32). An interim conference report of the breast cohorts is available, but its mixed HER2-expressing population does not justify an unqualified TNBC-specific efficacy claim (33). Separately, BEGONIA arm 6 reported T-DXd plus durvalumab in hormone-receptor-negative, HER2-low advanced disease. The reported response rate was 62.1% and did not meet the protocol-specified response objective. This illustrates why an apparently substantial response rate is not equivalent to a positive study. The regimen remains distinct from T-DXd-pembrolizumab and should be named accurately (34).

The phrase “chemotherapy-free” is misleading for ADC regimens carrying cytotoxic payloads. “Without conventional chemotherapy” can describe a trial backbone when appropriate, but it does not imply absence of cytotoxic exposure or reduced toxicity. ADC-ICI combinations should be evaluated using comparative adverse events, discontinuation, patient-reported outcomes and disease control rather than an assumed class-wide therapeutic advantage.

### 4.3 Antigen abundance and payload sensitivity are separate determinants

An ADC requires a sequence of events: access to the lesion, target binding, delivery of an active payload and susceptibility of the exposed cells. Failure at one step cannot necessarily be overcome by increasing another. The ASCENT exploratory biomarker analysis examined Trop-2 expression and germline BRCA1/2 status, providing a framework for studying variation in SG activity. It did not create a universally validated Trop-2 threshold for excluding patients from treatment. Small biomarker subgroups, archival sampling and measurement variability limit conclusions about a low-expression boundary. In particular, an ADC target-abundance measurement should not be treated as a surrogate for PD-L1 eligibility or immune competence (35).

Direct clinical resistance observations strengthen this distinction. Coates and colleagues identified parallel alterations involving antigen and payload targets during acquired resistance to SG in TNBC. These findings show that progression may involve the delivery target or the topoisomerase pathway targeted by the payload, and that resistance can be polyclonal. They do not establish the prevalence of each mechanism in an unselected population or guarantee resistance to every subsequent ADC. The appropriate translational question is whether a new target, linker or payload actually bypasses the measured defect. Adding checkpoint blockade does not automatically solve defective payload delivery or altered payload susceptibility (36).

A combination-development program should therefore measure both the tumor-killing and immune hypotheses. Where feasible, pretreatment and progression samples could characterize target expression, relevant genomic changes and spatial immune features. A payload-related change could support investigation of a different cytotoxic class; an immune recognition defect could motivate a distinct immune strategy. These are prospective hypotheses, not established sequencing instructions. This distinction is especially important when two ADCs share topoisomerase-I inhibition, because changing the surface target may leave downstream resistance unresolved. The retrospective sequential-ADC evidence discussed below is consistent with limited benefit in some patients, but cannot establish a single cross-resistance mechanism (36, 37).

### 4.4 What a convincing immune contribution would look like

Evidence of immunogenic cell death is stronger when it includes functional consequences rather than isolated increases in damage-associated molecules. The experimental model should distinguish stress responses from an immune response capable of recognizing and controlling tumor cells. An appropriate comparison might assess whether treatment benefit changes when a relevant immune population is absent, whether antigen-specific responses expand, and whether a second tumor challenge is controlled. Such experiments address mechanism in a model; they cannot substitute for randomized clinical evidence. Conversely, a clinical PFS advantage validates a regimen in its tested population without necessarily validating the proposed immune mechanism (26, 27).

For clinical translation, an integrated design could link paired biopsies to prespecified endpoints such as intratumoral localization of CD8 cells, antigen-presentation measurements and T-cell clonotype changes. Analyses should include nonresponders and should avoid selecting the sampling time retrospectively because it produces the largest difference. If only patients who remain well enough for a second biopsy contribute specimens, apparent immune activation may be biased toward favorable outcomes. Reporting biopsy availability and reasons for missing samples is therefore part of interpreting the biological result. A convincing immune contribution requires coherence between the randomized comparison, the timing of biological change and the proposed pathway, while acknowledging alternative explanations.

## 5 Biomarkers linked to specific treatment decisions

### 5.1 Retain PD-L1 while defining what an additional marker must improve

PD-L1 is imperfect, but imperfection does not establish that an alternative is ready to replace it. For first-line pembrolizumab combinations in advanced TNBC, the relevant evidence remains linked to the assay, scoring algorithm and clinical threshold used for treatment selection. For early-stage disease, the different eligibility framework must be retained. A candidate marker should therefore be evaluated against a specified decision: selecting an ICI-containing regimen, choosing its partner, detecting residual disease or identifying a resistance mechanism (2, 16, 23).

Preanalytical quality, specimen timing, tissue adequacy and the anatomical source should be recorded. An archival primary tumor and a recently sampled metastatic lesion may represent different treatment histories. However, heterogeneity alone does not justify a blanket requirement for repeat biopsy before every ICI decision. Reassessment should address a clinically relevant uncertainty and use a validated assay. **Supplementary Table 2** separates established selection criteria from research measurements.

### 5.2 Stromal lymphocytes and spatial organization

High stromal tumor-infiltrating lymphocyte levels can identify a favorable prognosis even without systemic chemotherapy. A pooled analysis of 1,966 patients with early TNBC treated with locoregional therapy but no chemotherapy provides direct evidence for this prognostic association. It cannot determine the additional benefit of an ICI or establish a safe treatment-omission threshold. Standardized scoring and clear reporting of the denominator are essential when comparing studies (38).

Spatial measurements may add information about whether effector cells reach malignant cells. Wang and colleagues examined spatial predictors in TNBC within a clinical trial context, linking cellular states and relationships to immunotherapy response. A trial-associated research analysis nevertheless requires further validation before it can function as a clinical decision rule. External validation, prespecified thresholds and demonstration of benefit from acting on the result are still required (3).

Tertiary lymphoid structures (TLS) add a further dimension through organized B-cell, T-cell and antigen-presenting-cell compartments. Foundational evidence linking B cells and TLS to checkpoint response includes melanoma and renal-cell carcinoma cohorts. These studies support biological investigation in TNBC; they do not validate a universal TNBC TLS threshold. Density, maturity, location and sampling variability should be defined separately, rather than treating any lymphoid aggregate as a functionally equivalent structure (39).

The International TILs Working Group recommendations provide the measurement foundation for stromal TIL assessment. The relevant denominator is the stromal area within the defined tumor boundaries, rather than the percentage of all cells or the number of lymphocytes in a selected hotspot. Scoring should be based on appropriate viable areas and should describe specimen type and handling. These distinctions are consequential: an automated density per square millimeter and a pathologist’s stromal percentage need not represent the same variable even when both are called a TIL score. Harmonization begins with the biological and pathological definition, not with a common numerical scale (40).

The prognostic evidence also spans different treatment contexts. A pooled individual-patient analysis evaluated TILs in chemotherapy-treated early TNBC, while a population-based study examined young, node-negative patients without systemic treatment. Their findings complement the larger untreated pooled analysis by showing why baseline immune infiltration can be associated with outcome independently of checkpoint blockade. None randomizes treatment according to a TIL threshold. Extrapolation from a low-risk, node-negative subgroup to stage III disease would also alter the absolute recurrence risk that a de-escalation decision must address (38, 41, 42).

Spatial assays may improve interpretation by separating stromal accumulation from contact with malignant nests, but increased assay complexity introduces additional sources of variability. The selected tissue region, cell segmentation, marker panel and definition of a cellular neighborhood can change the output. Keren’s imaging study established the relevance of organized tumor-immune architecture, whereas the trial-associated spatial predictor study more directly linked measurements to an immunotherapy setting. These are complementary levels of evidence. Before a spatial model informs care, its assay and computational pipeline should be fixed and tested in external specimens, including small biopsies and samples collected at different centers (3, 5).

A pooled neoadjuvant analysis of 3,771 patients across breast cancer subtypes additionally showed that the prognostic meaning of lymphocytic infiltration varies by subtype. This argues against transferring a TIL-outcome relationship indiscriminately from hormone-receptor-positive disease or treating all breast tumors as one validation cohort. It also reinforces the need to distinguish association with chemotherapy response from prediction of the additional benefit of an ICI. The population and comparator are part of a biomarker’s meaning (43).

### 5.3 Genomic and transcriptomic measurements

Germline BRCA1/2 alterations have established therapeutic relevance for PARP inhibition in appropriate HER2-negative breast cancer settings. They are not interchangeable with a genomic scar measure of homologous recombination deficiency, and neither automatically predicts an added benefit from checkpoint blockade. OlympiAD and OlympiA support particular uses of olaparib; they do not establish that adding an ICI improves those results (44, 45).

The DORA trial investigated olaparib with or without durvalumab as maintenance after benefit from platinum treatment. It was a small, randomized but noncomparative phase II trial, and it included a broader receptor-expression definition than strict ER/PR-negative TNBC. Its design and selection for prior platinum benefit limit claims about incremental durvalumab efficacy or activity in platinum-refractory disease. It should be interpreted as hypothesis-generating maintenance evidence (46).

The FDA’s tissue-agnostic TMB-high indication for pembrolizumab specifies unresectable or metastatic solid tumors with ≥10 mutations per megabase, as determined by an FDA-approved test, after prior progression and when no satisfactory alternative treatment options are available (47). The FDA companion-diagnostic list identifies authorized tests and their corresponding intended uses (48). These regulatory sources do not validate exploratory TNBC transcriptomic classifiers as first-line alternatives to PD-L1 testing.

Transcriptomic immune classifiers require a separate validation framework. The assay, gene set, scoring algorithm and decision threshold should be fixed before independent validation, with a prespecified analysis plan. Predicting differential treatment benefit requires an appropriate treatment comparison, generally from a randomized trial, and an analysis designed to evaluate treatment effects by biomarker status (49). During model development, gene selection and model fitting must occur within each training fold; selecting genes using the full dataset before cross-validation can bias performance estimates (49, 50). These methodological principles do not establish clinical utility for any particular TNBC immune signature.

Genomic scar measures require particular caution because they summarize accumulated consequences of defective repair rather than necessarily measuring current repair function. In an exploratory OlympiAD analysis, olaparib activity was observed irrespective of the HRD score in patients already selected by germline BRCA mutation. Thus, layering an additional scar threshold onto an established germline-defined indication can exclude patients without a validated reason. Conversely, a high scar score in a germline-wild-type tumor does not by itself establish sensitivity to an ICI-PARP combination. The assay, genetic context and treatment question must be specified together (105).

Transcriptomic classifiers face a related compartment problem. Refinement of TNBC molecular subtypes showed how infiltrating immune and stromal cells contribute to bulk expression. An “immune-high” classification can therefore reflect abundance, activation or tissue composition, with different implications for intervention. NeoPACT’s evaluation of predefined immune expression signatures is useful because it identifies specific assays and a specific treatment cohort, rather than an unspecified future multi-omics score. However, association with pCR under one regimen does not isolate pembrolizumab’s added effect or establish that an alternative treatment would perform differently. Comparative validation should retain the classifier and threshold established before outcome analysis (4, 14). The prespecified validation framework is described by Simon and colleagues (49).

### 5.4 ctDNA and the gap between prognosis and actionable residual disease

ctDNA detection can identify molecular residual disease and provide serial information unavailable from a single biopsy. The decisive question is whether changing treatment because of a result improves outcomes. In c-TRAK TN, 161 patients entered surveillance; among patients allocated to intervention, 72% had metastatic disease on staging when ctDNA was detected. Five patients commenced pembrolizumab, and none achieved sustained ctDNA clearance. The small treated group and the frequency of already visible metastases limited evaluation of early intervention (9).

These findings support a distinction between prognostic validity and clinical utility. Currently, no prospective interventional evidence supports omitting the adjuvant pembrolizumab component of a KEYNOTE-522-type regimen solely because ctDNA is negative. A negative result means that tumor-derived DNA was not detected in that sample by that assay; it does not establish absence of residual cancer or absence of benefit from continued immunotherapy. This limitation remains relevant after repeated negative measurements, particularly when pretreatment ctDNA was undetectable or tumor shedding is low. Conversely, ctDNA positivity does not identify an effective escalation regimen by itself. Neither treatment omission nor an untested ADC–ICI escalation should therefore be based on ctDNA status alone outside an appropriate clinical trial (8, 9, 51–54).

Routine ctDNA-MRD implementation is constrained by differences between tumor-informed and tumor-agnostic platforms, variant coverage, analytical sensitivity, sample requirements and definitions of positivity. Low residual tumor burden, limited shedding and preanalytical losses can produce false-negative results, whereas clonal hematopoiesis can produce plasma variants unrelated to the tumor. Paired leukocyte analysis, where appropriate to the assay, can help address the latter problem but does not resolve false negatives. Additional barriers include tumor-tissue availability for personalized assay development, repeated phlebotomy, specimen transport and processing, turnaround time, cost and reimbursement. A harmonized routine-care sampling schedule for TNBC has not been established, and results obtained using different platforms and sampling intervals should not be treated as interchangeable (9, 51, 54, 55).

Missing serial samples are a separate source of uncertainty. Patients who relapse early, become unwell, discontinue follow-up or face travel and financial barriers may be less likely to provide repeated blood samples. Analyses restricted to patients with complete longitudinal sampling can therefore select a healthier or better-resourced subgroup and distort estimates of recurrence risk and assay performance. Studies should report the eligible, sampled and successfully tested denominators at each time point, reasons for missingness, and differences between patients with and without serial samples. Missing or failed tests must not be classified as negative. Prespecified landmark or longitudinal analyses should address the timing of sample availability, with sensitivity analyses for informative missingness; statistical adjustment cannot guarantee removal of selection bias.

The ctDNA evidence should be read as a progression from detection to clinical utility. Personalized assays have identified molecular relapse before clinically apparent breast cancer recurrence, as shown in the Coombes study. The BRE12–158 secondary analysis provided disease-specific evidence that postoperative ctDNA and circulating tumor-cell findings were associated with recurrence in residual TNBC. These studies establish that blood-based measurements can stratify risk, but they do not demonstrate that an intervention prompted by the result changes that risk. The distinction remains even when the association is strong or the lead interval is clinically attractive (52, 53).

Longer follow-up adds useful information but also emphasizes assay and population dependence. Shaw and colleagues monitored 156 patients with breast cancer using prospectively collected samples analyzed retrospectively; their study included a relatively small TNBC subgroup. The observations support sustained prognostic value, but the study did not compare ctDNA-triggered treatment with usual surveillance. Serial negative results should also be interpreted relative to baseline detectability and sampling frequency. A patient whose tumor sheds little detectable DNA presents a different surveillance problem from one with a readily measurable pretreatment signal (54).

False-positive and false-negative results lead to different harms. Hu and colleagues demonstrated in lung cancer that some plasma variants originate from clonal hematopoiesis rather than the tumor. This is indirect cancer-type evidence for a general analytical problem, supporting consideration of paired leukocyte sequencing and careful interpretation of variants in hematopoiesis-associated genes. On the other side, low tumor burden and limited shedding may prevent detection despite residual disease. For an escalation trial, a false-positive result can expose a patient to unnecessary toxicity; for an omission trial, a false-negative result can remove effective treatment. Validation must therefore reflect the intended decision, rather than reporting a single accuracy statistic (55).

BRE12–158 further illustrates why molecular characterization should not be equated with successful personalization. Its genomically directed postneoadjuvant strategy was not superior to treatment of physician’s choice, even though ctDNA remained prognostic. A platform can identify abnormalities accurately yet fail to improve outcomes if the available intervention is ineffective, arrives too late or targets a nondominant process. This negative result belongs beside positive biomarker associations when evaluating clinical utility. It supports testing the entire measurement-to-treatment strategy, including turnaround time, treatment availability and adherence (56).

### 5.5 Composite models and microbiome measurements

A composite signature should be developed for a prespecified population, decision time and clinical action. Any future model must demonstrate incremental value beyond a credible baseline containing standard clinicopathological variables and PD-L1 status where relevant to the treatment setting. Depending on the intended use, baseline variables may include disease stage, nodal status, performance status, prior treatment and, for postoperative decisions, pathological response or residual cancer burden. Improvement in retrospective discrimination alone is insufficient: evaluation should include calibration, clinical net benefit and external performance. If the signature is intended to select an ICI-containing regimen, improved prediction of recurrence or response must not be confused with prediction of differential treatment benefit; the latter requires an appropriate comparator and a prespecified treatment-by-biomarker analysis. Inclusion of PD-L1 in research comparisons does not make it an eligibility requirement for the KEYNOTE-522 regimen (49, 60).

Microbiome measurements remain particularly sensitive to diet, medications, geography, specimen handling and contamination. Interventional fecal microbiota transplantation evidence in anti-PD-1-resistant melanoma supports investigation of host effects but cannot establish benefit in TNBC. Routine microbiome-directed treatment selection, probiotics or fecal transplantation cannot be inferred from these data. In **Figure 2**, microbiome information is therefore included as a candidate research input, with no claim that a validated “Spatial Immune Score” replaces standard testing (57).

**Figure 2:**
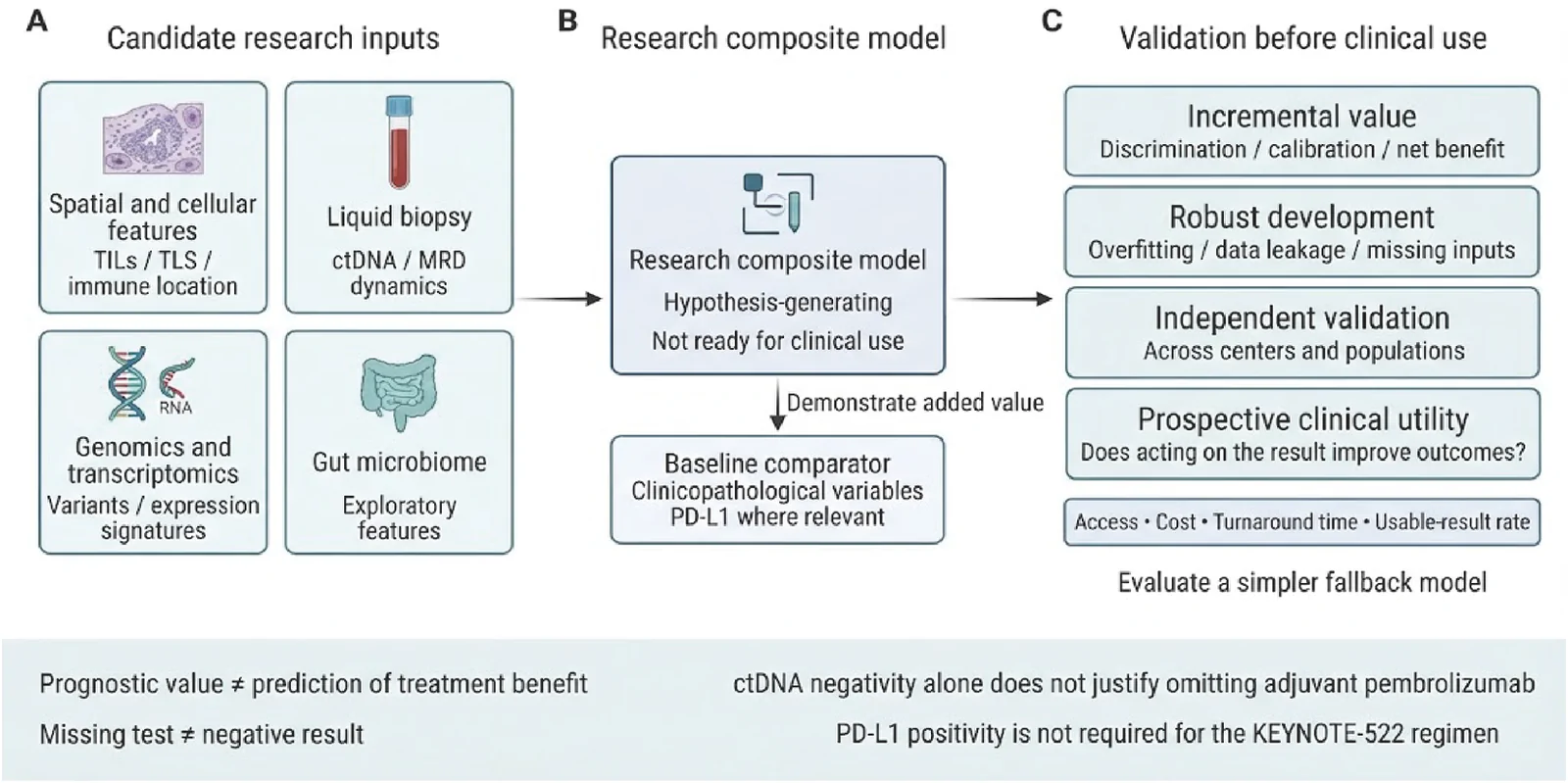
Hypothesis-generating biomarker domains and boundary conditions for composite-model research. **(A)** Candidate research inputs: spatial and cellular features (TILs, TLS, immune location), liquid biopsy (ctDNA/MRD dynamics), genomics and transcriptomics (variants, expression signatures), and gut microbiome (exploratory features). **(B)** Research composite model: a conceptual, hypothesis-generating framework (not a validated score or clinical tool) that must demonstrate added value beyond baseline clinicopathological variables and setting-appropriate PD-L1 assessment. **(C)** Validation before clinical use: required steps include incremental value assessment (discrimination, calibration, net benefit), robust development (overfitting protection, data leakage prevention, missing input handling), independent validation across centers and populations, and prospective clinical-utility testing. Feasibility evaluation must address assay access, cost, turnaround time, and performance when advanced measurements are unavailable; a simpler fallback model should be evaluated. Key boundary conditions: Prognostic value ≠ prediction of treatment benefit; ctDNA negativity alone does not justify omitting adjuvant pembrolizumab; missing test ≠ negative result; PD-L1 positivity is not required for the KEYNOTE-522 regimen (3, 9, 16, 49, 50, 60).

Microbiome evidence additionally requires separation by specimen and disease. Routy and colleagues linked gut microbiome features to PD-1-based immunotherapy in epithelial tumors, while Spencer and colleagues examined diet, probiotic use and microbiome features in melanoma alongside preclinical experiments. These studies motivate mechanistic hypotheses, but neither defines a TNBC prescribing algorithm. Stool profiles should not be relabeled as intratumoral microbial composition, and an observational association with dietary intake cannot establish that prescribing a supplement will improve ICI efficacy. Antibiotic exposure may also mark infection severity and other clinical differences, so an association should not lead to withholding indicated antimicrobial treatment (58, 59).

For composite models, the relevant comparison is not only one complex model against another. Investigators should show whether new measurements improve a clinically credible baseline model, assess calibration across risk levels and explain the consequence of a positive result. TRIPOD+AI provides a reporting framework for prediction models, including clear specification of development and evaluation. If an AI-supported decision is tested as an intervention, CONSORT-AI addresses a different stage: how the intervention is used and evaluated within a clinical trial. Compliance with reporting guidance does not validate a TNBC model, but it makes the evidence assessable and the workflow reproducible (60, 61).

High-dimensional integration creates a substantial risk of overfitting when the number of candidate features is large relative to the number of patients and outcome events. Feature selection, imputation and model tuning must occur within the training process, and evaluation must separate patients rather than individual image tiles or repeated blood samples. The final assay workflow, model and decision threshold should be fixed before independent validation. Missing modalities are likely in routine care because of inadequate tissue, failed assays, missed blood draws or limited local availability; these causes may be associated with prognosis and access to care. Investigators should prespecify missing-data handling, report performance and coverage when modalities are unavailable, and evaluate a simpler fallback model rather than relying only on complete-case results. Many centers lack access to spatial immune profiling, specialized tissue processing and computational support. Cost, turnaround time and the proportion of patients for whom a usable result can be generated are therefore essential feasibility endpoints, not secondary implementation details. Advanced profiling remains a research activity and should not delay established testing or treatment (50, 60).

## 6 Mechanism directed interventions for immune exclusion and resistance

### 6.1 Define the failure before selecting an intervention

Primary nonresponse and acquired progression after initial benefit should be recorded separately, including the timing of progression, prior ICI exposure and sites of disease. Lack of response to a combination may reflect resistance to the cytotoxic partner, immune failure, inadequate exposure or several factors together. Spatial immune-desert, excluded and inflamed patterns provide biological descriptions, but none supplies a self-sufficient treatment prescription (**Figure 3**).

**Figure 3:**
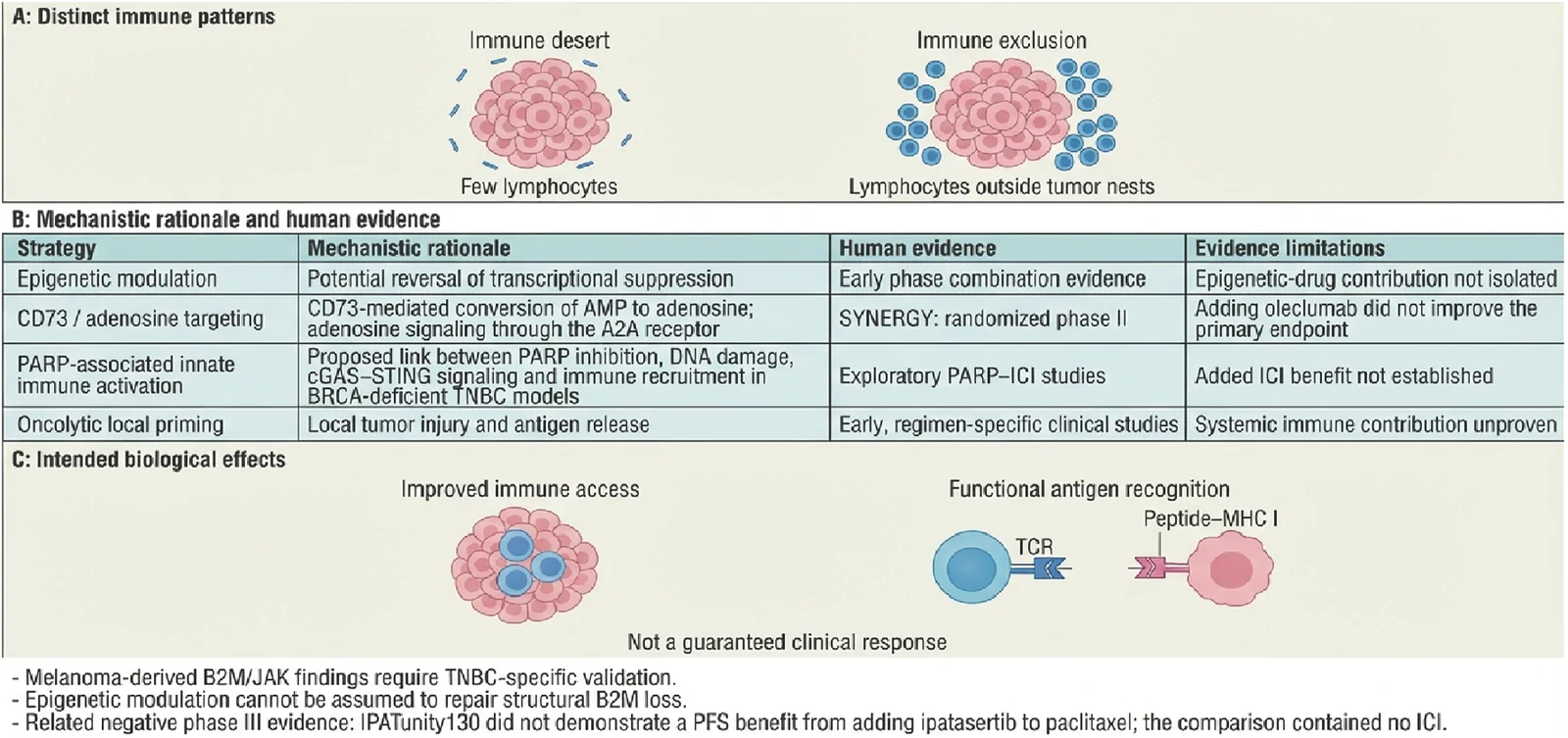
Investigational strategies for immune-desert and immune-excluded tumors: mechanistic rationale and clinical evidence boundaries. **(A)** An immune-desert pattern contains few lymphocytes, whereas an immune-excluded pattern contains lymphocytes predominantly outside malignant cell nests. These spatial patterns do not independently identify a validated treatment strategy. **(B)** Four investigational approaches are summarized alongside human evidence and limitations: epigenetic modulation of potentially reversible transcriptional suppression; targeting of CD73-mediated adenosine production or downstream A2A-receptor signaling; PARP-associated innate immune activation supported by BRCA-deficient TNBC models; and oncolytic virus-mediated local tumor injury and antigen release. Early clinical combination studies do not isolate the contribution of epigenetic treatment. PARP–ICI and oncolytic studies provide exploratory evidence without establishing an incremental ICI benefit or a causal systemic immune contribution, respectively. In the randomized phase II component of SYNERGY, adding the anti-CD73 antibody oleclumab to durvalumab, paclitaxel and carboplatin did not improve the primary endpoint. This result applies to the tested regimen and does not establish failure of every adenosine-directed strategy. **(C)** Improved intratumoral immune access and TCR recognition of tumor peptide–MHC class I complexes illustrate intended biological effects, not demonstrated outcomes of all depicted interventions or a guaranteed clinical response. The mechanistic descriptions represent investigational hypotheses rather than established treatment effects in human TNBC. Foundational B2M/JAK resistance findings from melanoma require TNBC-specific validation, and epigenetic modulation cannot be assumed to repair structural B2M loss. As related negative randomized evidence, phase III IPATunity130 did not demonstrate a progression-free survival benefit from adding ipatasertib to paclitaxel; that comparison contained no ICI and was not a test of checkpoint-resistance reversal. The image is a research framework, not a clinical treatment algorithm (46, 63, 67, 69–72, 75–77, 80, 81). TNBC, triple-negative breast cancer; ICI, immune checkpoint inhibitor; PARP, poly(ADP-ribose) polymerase; TCR, T-cell receptor; MHC, major histocompatibility complex; B2M, β2-microglobulin; JAK, Janus kinase; cGAS, cyclic GMP–AMP synthase; STING, stimulator of interferon genes.

WNT/β-catenin signaling, impaired interferon responses and defective antigen presentation provide candidate explanations for immune resistance, but their supporting evidence differs by tumor type. Foundational observations of acquired JAK1/JAK2 and B2M alterations during PD-1 blockade came from melanoma, including a small paired-biopsy cohort. These findings do not establish the prevalence, functional importance or treatment-predictive value of the same alterations in TNBC. A detected variant also does not automatically establish pathway loss or identify the cause of progression on a combination regimen. TNBC-specific studies should integrate pretreatment and progression specimens, allelic status, protein expression and functional measurements before these alterations are used to select a resistance-directed intervention (62, 63).

Reversible transcriptional suppression must also be distinguished from a structural loss of function. An epigenetic drug might increase expression of a silenced pathway, but it cannot be assumed to repair a biallelic B2M deletion or restore a deleted gene. Consequently, **Supplementary Table 3** avoids pairing every antigen-presentation defect with HDAC/DNMT inhibition as though the same rescue strategy applied. DNA findings, protein expression and functional immune measurements may need to be considered together in a research setting.

Stromal exclusion deserves its own analysis because simply increasing circulating T-cell numbers may not bring them into contact with cancer cells. Human breast cancer studies have identified distinct fibroblast populations with different relationships to immune suppression. The TGFβ-associated exclusion model was developed using urothelial cancer specimens and experimental systems, providing a rationale for stromal intervention but not proof of benefit in TNBC. Similarly, the foundational β-catenin experiment used melanoma. These findings can inform a TNBC trial only after establishing that the relevant process is present and functionally important in the selected tumors (64–66).

A useful experimental strategy would distinguish failure of recruitment from failure of recognition. Sparse intratumoral lymphocytes could prompt investigation of vascular, chemokine or stromal barriers. Infiltrating cells without tumor recognition could prompt examination of antigen presentation and receptor specificity. An inflamed tumor that still progresses might require analysis of suppressive myeloid cells, dysfunctional effector states or resistant malignant clones. Single-cell breast cancer studies support the existence of heterogeneous cellular responses, but no one of these patterns is yet a sufficient clinical prescription. The framework therefore narrows the hypothesis to be tested rather than offering a menu of automatic rescue regimens (6, 7).

### 6.2 Epigenetic modulation and antigen density

Epigenetic priming is based on the hypothesis that selected transcriptional defects may be reversible at tolerable drug exposure. Its relevance depends on the substrate required by the accompanying immune therapy. In the AML experiments reported by Cheng and colleagues, decitabine and chidamide increased CD70 expression and enhanced CD70-directed CAR-T cytolysis. These findings concern a surface antigen recognized by a specific CAR product; they do not establish restoration of peptide–HLA presentation in TNBC. The reported antigen-density threshold and fold changes are specific to the experimental system and should not be transferred to breast cancer or other cellular products. Epigenetic modulation also cannot be assumed to repair structural loss such as a biallelic B2M deletion (68).

Human breast cancer evidence remains preliminary. A phase Ib study evaluated entinostat with nivolumab and ipilimumab in 24 women with advanced HER2-negative breast cancer, including 12 with TNBC. Responses occurred in four of ten evaluable TNBC participants, but safety was the primary endpoint and the small, uncontrolled combination study could not isolate the contribution of entinostat. It therefore does not establish that epigenetic priming restores ICI sensitivity in immune-desert or ICI-resistant TNBC (67).

The next translational step is to identify a measurable, reversible defect in TNBC and show that a specific epigenetic agent corrects it at an acceptable exposure. Paired specimens should distinguish target engagement from restored immune function, and an appropriate comparator should determine whether the epigenetic component improves clinical outcomes. Antigen abundance, HLA-dependent recognition and CAR-mediated recognition should remain separate endpoints rather than interchangeable indicators of immune rescue.

### 6.3 Adenosine and oncogenic signaling

The adenosine hypothesis is supported by preclinical breast cancer studies in which CD73 targeting affected tumor growth and metastasis, and by TNBC-focused work linking CD73 to anthracycline resistance through an immune mechanism involving adenosine signaling. These observations provide a rationale for intervention, but do not establish that CD73 expression identifies a clinically actionable dependence in human TNBC (71, 72).

The randomized phase II component of SYNERGY directly tested one clinical implementation: adding oleclumab to durvalumab, paclitaxel and carboplatin in previously untreated advanced TNBC. The trial did not meet its primary endpoint; 24-week clinical benefit rates were 43% with oleclumab and 44% without it. This result does not exclude every adenosine-directed approach, but it does not support routine addition of oleclumab to the tested backbone. Further studies should justify how their population, drug, schedule or functional biomarker addresses the limitations exposed by this negative comparison, rather than treating preclinical activity as sufficient evidence of clinical benefit (69).

Oncogenic-pathway targeting requires the same separation of rationale and clinical validation. In biomarker-selected advanced TNBC, the phase III IPATunity130 trial did not demonstrate improved progression-free survival from adding ipatasertib to paclitaxel. This comparison did not include an ICI and therefore was not a test of checkpoint-resistance reversal. PIK3CA/AKT1/PTEN alteration status alone should not be treated as a validated indication for an AKT inhibitor–ICI combination. Future immune-focused trials require evidence of the relevant suppressive phenotype, pharmacodynamic pathway modulation and an appropriate treatment comparison (70).

Adipocyte-derived CCL5 and insulin-resistance-associated exosomes provide additional experimental links between the metabolic microenvironment and TNBC behavior. However, effects on proliferation, invasion or metastasis do not by themselves demonstrate an immune mechanism. The studies cited here do not establish a TNBC clinical strategy for restoring checkpoint sensitivity through emodin or exosome-directed treatment. Further work should measure immune consequences directly and distinguish them from tumor-cell-intrinsic effects before proposing an ICI-resistance intervention (73, 74).

### 6.4 DNA damage and innate immune sensing

In BRCA-deficient TNBC models, PARP inhibition can activate intratumoral STING signaling and recruit CD8-positive T cells. This provides disease-relevant preclinical support for an immune-priming hypothesis, but neither a BRCA alteration nor an HRD genomic scar establishes that the relevant sensing pathway remains functional in a human tumor. PARP inhibitors also have direct antitumor activity, creating an alternative explanation for responses observed during combination treatment (75).

Human studies provide activity signals without definitive attribution of an added ICI benefit. TOPACIO evaluated niraparib plus pembrolizumab in advanced TNBC using a single-arm design. MEDIOLA evaluated olaparib followed by olaparib plus durvalumab in germline BRCA-mutated, HER2-negative metastatic breast cancer, a population broader than TNBC. DORA investigated olaparib with or without durvalumab after benefit from platinum therapy, but its small randomized, noncomparative phase II design was not a definitive test of incremental durvalumab efficacy. These studies differ in disease definition, mutation selection, prior platinum sensitivity and treatment timing and should not be presented as interchangeable demonstrations of immunological synergy (46, 76, 77).

Future trials should retain an appropriate PARP-based comparator, prespecify prior platinum and PARP exposure, and test whether a functional immune or DNA-sensing measurement identifies differential benefit from adding an ICI. Tumor and host pathway activity should be assessed separately where feasible. Changes in interferon-related transcripts or lymphocyte infiltration can support mechanistic interpretation, but cannot replace comparative evidence for disease control, survival and toxicity.

### 6.5 Local immune priming and alternative checkpoint approaches

Local immune priming proposes that tumor injury, antigen release and inflammatory signaling may increase susceptibility to checkpoint blockade. For radiotherapy and oncolytic viruses, however, local cytotoxicity must be distinguished from systemic tumor-specific immunity. Antigen release alone does not demonstrate effective cross-presentation, recruitment of functional T cells or control of distant lesions.

Human TNBC evidence remains exploratory. TONIC evaluated short induction strategies followed by nivolumab in a 67-patient randomized but noncomparative phase II study; it was not designed to establish a definitive ranking of induction regimens. A separate small phase II study evaluated radiotherapy plus pembrolizumab. These studies support continued investigation but do not establish a reproducible abscopal treatment effect or a standard method of reversing ICI resistance (78, 79).

For oncolytic therapy, the phase II TNBC study of T-VEC combined the virus with neoadjuvant chemotherapy and did not test an ICI-containing regimen. PROMETEO subsequently evaluated T-VEC plus atezolizumab in residual HER2-negative breast cancer, including TNBC and selected hormone-receptor-positive disease. Its single-arm design and mixed population limit attribution of benefit to the virus, the ICI or their interaction. Future studies should define the injectable disease setting, distinguish injected from noninjected lesion responses, incorporate appropriate controls and evaluate clinical outcomes beyond local immune changes (80, 81).

Alternative checkpoint strategies also require regimen-specific interpretation. The nonrandomized adaptive phase II BELLINI study provides early human TNBC evidence for nivolumab with or without ipilimumab, but does not establish routine chemotherapy omission or reversal of acquired metastatic ICI resistance. The effects and toxicity of this checkpoint pair cannot be transferred to LAG-3, TIGIT or TIM-3 blockade. Comparative evaluation and prospectively defined patient selection remain necessary (82).

### 6.6 Cellular therapies as a limited cross disease comparison

Engineered cellular therapies face product-specific questions about antigen density, target heterogeneity and effects on normal tissues. Their relevance here is to illustrate the need for a defined target and safety framework, rather than to expand this review into a survey of cellular therapy. In Cheng and colleagues’ phase I trial, CD5-knockout anti-CD5 CAR-T cells were administered to seven patients with relapsed/refractory CD5-positive hematological malignancies. Six responded, including four complete responses; cytokine release syndrome occurred in all seven and neurotoxicity in two. Cytopenias, infections and prolonged lymphocyte depletion were important observations (83).

This dataset is not TNBC efficacy evidence and does not demonstrate activity against visceral breast cancer metastases. It illustrates why the normal-tissue distribution of a target and the biology of a cellular product determine toxicity. Such risks should not be conflated with checkpoint-related endocrinopathy or ADC payload toxicity. A future TNBC cellular-therapy study would need its own antigen-selection, manufacturing and safety evidence before outcomes from hematological disease could inform expectations.

### 6.7 Tumor specific T cells and individualized vaccination

Cellular immunotherapy should also be represented by breast cancer evidence rather than only by hematological examples. Zacharakis and colleagues reported durable complete regression in a patient with hormone-receptor-positive metastatic breast cancer treated using mutation-reactive tumor-infiltrating lymphocytes with additional treatment components. This was an important proof of concept for tumor-specific recognition in breast cancer, but it was neither a TNBC CAR-T trial nor a population-level efficacy estimate. It illustrates the importance of identifying actual tumor-reactive specificities instead of assuming that any expanded lymphocyte population will be therapeutically effective (84).

Individualized vaccines offer another way to address limited antigen-specific immunity. A phase I TNBC study administered personalized neoantigen DNA vaccines to 18 patients with residual disease after neoadjuvant treatment and demonstrated feasibility and neoantigen-specific immune responses. A 2026 report investigated individualized mRNA vaccination in 14 patients with TNBC following surgery and systemic therapy, providing evidence of durable vaccine-induced T-cell immunity. Both reports are directly relevant to TNBC vaccine development, but their early, noncomparative designs do not establish a reduction in recurrence attributable to vaccination. Immunogenicity, treatment feasibility and clinical efficacy are separate endpoints (85, 86).

The biological distinction from CAR-T therapy is important. A vaccine aims to induce or expand immune responses to selected antigens, and those responses depend on antigen processing, presentation and a competent host immune repertoire. A CAR product supplies engineered recognition of a surface target. An HLA-pathway defect could therefore have different implications for these approaches. Vaccination also does not automatically overcome stromal exclusion: a measurable peripheral T-cell response must still translate into access to and recognition of residual cancer. This makes paired tissue and blood measurements valuable in developmental studies, while avoiding the inference that circulating immunogenicity is a validated surrogate for recurrence prevention.

Manufacturing time, availability of adequate tumor material and patient-specific antigen selection are additional elements of clinical utility. A personalized product that cannot be delivered during the relevant treatment window may be biologically active but operationally unsuitable. Future trials should report the denominator of patients screened, successfully manufactured and treated, together with reasons for failure or delay. The clinical comparison should reflect the contemporary postoperative treatment context rather than historical cohorts that received materially different therapy. Such information would connect the vaccine’s biological promise to an implementable intervention without broadening this review into an unrelated catalogue of cell products.

## 7 Translating biomarker hypotheses into feasible care

### 7.1 Treatment sequencing and remaining clinical uncertainty

Current treatment selection should start with the disease setting, prior therapy, biomarker-defined eligibility and local availability. For advanced TNBC with CPS ≥10, the United States now has first-line SG-pembrolizumab approval in addition to the established pembrolizumab-chemotherapy evidence. For patients ineligible for PD-1/PD-L1-based treatment, SG monotherapy has a distinct first-line indication. These categories should not be merged into a recommendation to use an ICI for all patients receiving an ADC (23).

Prior perioperative immunotherapy creates an important uncertainty at recurrence. A first-line trial result in its specified population does not automatically establish benefit from rechallenge after early ICI-exposed relapse. Future studies should stratify by previous ICI use and treatment-free interval. Clinical sequencing also requires attention to the activity remaining after exposure to ADCs with related payload classes. A retrospective study of sequential SG and T-DXd in HER2-low metastatic breast cancer suggests that benefit from a subsequent ADC can be limited, but it cannot identify an optimal sequence or eliminate confounding by treatment line and patient selection (37).

For patients with germline BRCA1/2 alterations, established PARP inhibitor evidence should be considered on its own terms. Neither an HRD score nor ctDNA persistence is sufficient to prescribe an untested PARP inhibitor-ADC-ICI combination. In early disease, uncertainty about how best to integrate post-neoadjuvant treatments should be made explicit rather than resolved through unsupported assertions of synergy (45, 46).

Post-neoadjuvant sequencing must integrate several evidence streams that were not tested as one regimen. CREATE-X established a role for capecitabine in HER2-negative residual invasive disease after preoperative chemotherapy, while OlympiA established adjuvant olaparib in eligible high-risk germline BRCA-associated HER2-negative disease. Neither trial established the efficacy and safety of combining its intervention with the complete modern KEYNOTE-522 strategy. Thus, several individually supported treatments do not automatically constitute a validated concurrent combination. The practical uncertainty concerns selection, overlap and timing, and should be acknowledged rather than resolved by assuming additive efficacy (1, 45, 87).

For clinical research, residual burden, germline BRCA status, prior immune exposure and treatment tolerance should be recorded as distinct variables. The residual-burden analysis informs prognosis after neoadjuvant treatment, whereas germline BRCA status can identify a specific targeted-therapy indication. ctDNA can identify another risk dimension, but its presence does not determine whether capecitabine, olaparib, an ADC or an experimental immune strategy is best. A future trial could stratify on these variables while testing a defined treatment comparison. This approach preserves the value of each measurement without creating an unsupported treatment algorithm from their coexistence (10, 53).

### 7.2 Safety can invalidate an otherwise plausible combination

SG-related diarrhea and neutropenia, deruxtecan-associated pulmonary toxicity and checkpoint-related inflammation can create overlapping presentations. In DESTINY-Breast04, adjudicated drug-related interstitial lung disease/pneumonitis occurred in 12.1% of T-DXd recipients, including fatal events in 0.8%. These are monotherapy study estimates and must not be relabeled as the incidence for a T-DXd-ICI combination. Actual combination studies and product-specific guidance are needed to quantify risk (31).

When a patient develops diarrhea, pulmonary symptoms or organ dysfunction, the differential diagnosis includes treatment toxicity, infection, progression and unrelated disease. Investigation and treatment should be guided by severity. Severe suspected immune-related toxicity should not be left untreated while waiting for every infectious investigation to return; urgent evaluation and indicated corticosteroid treatment may need to proceed in parallel. Endoscopy, bronchoscopy or specialist referral should be used when clinically indicated rather than mandated for every presentation (88).

Safety evaluation should include dose interruptions, treatment discontinuation, hospitalization and persistent endocrine consequences. Distinct toxicities may require different interventions, and treatment of a serious immune-related event should not be withheld on an unproven assumption that immunosuppression necessarily abolishes antitumor efficacy. Patient-reported outcomes should accompany clinician grading when assessing the acceptability of prolonged or intensified therapy (88).

Organ-specific assessment remains essential even when several agents share a symptom. Diarrhea during SG-containing treatment may reflect payload toxicity, infection or immune-mediated colitis; pneumonitis during a deruxtecan combination may have drug-related or immune-mediated contributions. The distinction affects interruption, investigation and possible retreatment. Baseline symptoms, onset relative to dosing and severity should therefore be documented. ASCO and SITC guidance provides a framework for evaluating suspected immune-related adverse events, but it should be combined with the relevant product-specific guidance rather than used as a universal management pathway for every ADC toxicity (88, 89).

Endocrine events also illustrate why adverse-event counts alone do not capture burden. Persistent hormone replacement can matter long after anticancer treatment ends, while symptoms such as fatigue can be attributed incorrectly to chemotherapy or cancer. A safety assessment should distinguish transient laboratory abnormalities, symptomatic organ dysfunction and long-term treatment requirements. Severe cardiopulmonary or neurological presentations warrant urgent evaluation; a review should not imply that every possible alternative diagnosis must be excluded before treatment of a dangerous suspected immune event begins. These principles are relevant to treatment delivery even when the mechanism of combination efficacy remains uncertain (89).

Observational reports linking corticosteroid exposure with poor ICI outcomes require attention to indication and timing. Ricciuti and colleagues examined this issue in lung cancer and found that unfavorable associations differed between palliative and nonpalliative indications. This is not TNBC-specific evidence, but it demonstrates why illness severity can confound a drug-outcome association. Baseline steroids for symptomatic progressive cancer are not equivalent to steroids prescribed after an immune-related adverse event. Analyses should therefore distinguish baseline and time-varying exposure, reason for treatment and event severity. They cannot justify withholding indicated management for serious toxicity (88, 90).

Patient-reported outcomes provide a complementary measure of net benefit. The ASCENT quality-of-life analysis examined SG against chemotherapy in metastatic TNBC, allowing symptoms and functioning to be interpreted alongside the randomized efficacy result. Those findings belong to SG monotherapy in that trial, not automatically to SG-pembrolizumab. Combination studies should collect symptomatic toxicity, functioning, treatment burden and reasons for questionnaire noncompletion. If patients who deteriorate stop completing questionnaires, an apparently stable mean quality-of-life score among remaining participants can be misleading. Longitudinal reporting should make the denominator and missingness visible (91).

### 7.3 What real world evidence can establish

A retrospective cohort included 86 patients treated with the KEYNOTE-522 regimen and 154 controls; pathological complete response rates were 59.3% and 33.1%, respectively. There was no significant survival difference at a median follow-up of 2.4 years. This supports evaluation of implementation in practice, but the nonrandomized comparison is susceptible to differences in patient selection and treatment era. It does not prove a universal real-world survival decrement, nor can a cross-study comparison with a trial estimate establish that routine care attenuates the causal treatment effect (92).

Three methodological priorities would make future real-world TNBC studies more informative. First, comparative studies should prespecify the target trial being emulated, including eligibility, treatment strategies, a common time zero, follow-up, outcomes and the causal contrast. Disease stage, prior ICI and ADC exposure, performance status and treatment era should be recorded, with appropriate handling of time-varying treatment and confounding. This design discipline reduces avoidable bias but does not eliminate unmeasured confounding (93).

Second, biomarker studies should define the testing denominator before analysis. Reports should distinguish all patients clinically eligible for testing, those offered testing, those sampled, those with a valid result and those who initiated the biomarker-linked treatment. PD-L1 reporting should include assay, scoring algorithm, threshold, specimen site and timing. Untested patients, technically failed assays and unavailable results must remain distinct from biomarker-negative patients. Testing completion, turnaround time and treatment initiation are measurable implementation outcomes alongside efficacy (16, 97).

Third, missing biomarker results, serial samples and outcome follow-up should be reported by center and clinically relevant patient groups, with reasons for missingness where available. Analyses should compare included and excluded patients, prespecify methods for handling missing data and test sensitivity to informative missingness. Where observational data are used to investigate predictive biomarkers, the analysis should include an appropriate treatment comparator and evaluate treatment effects by biomarker status; outcomes within ICI-treated patients alone cannot establish a treatment-predictive effect (49, 93).

A tiered implementation strategy should prioritize the diagnostic information needed for established care. The minimum pathway includes reliable histopathological confirmation, quality-assured ER, PR and HER2 assessment, appropriate staging and evaluation of treatment fitness and baseline organ function. For advanced TNBC, PD-L1 testing should use the validated assay and scoring method required for the intended ICI regimen under local guidance; metastatic thresholds should not be imported into selection for the KEYNOTE-522 regimen. Germline BRCA1/2 testing should be available when indicated and relevant to an established treatment or hereditary-risk decision. Spatial immune profiling, broad multi-omics integration, microbiome assays and ctDNA-MRD testing to direct ICI escalation or omission should remain within prospective research. These research measurements should not delay treatment-defining diagnostics, indicated therapy or access to toxicity management (1, 2, 16, 44, 45, 88).

Routine-care evidence should additionally distinguish effectiveness from delivery failure. Failure to obtain a valid PD-L1 result, delayed treatment authorization, early discontinuation and biological progression are different events with different remedies. A cohort restricted to treated patients cannot quantify access among all eligible patients. Conversely, a lower unadjusted survival estimate may reflect case mix rather than reduced biological efficacy. Useful datasets should report testing completeness, treatment initiation, dose delivery and discontinuation reasons together with outcomes. The KEYNOTE-522 real-world cohort provides an implementation example, but broader conclusions about all health systems require broader data (92).

Economic interpretation should also keep cost-effectiveness separate from affordability. A regimen might produce additional quality-adjusted survival at an acceptable modeled ratio while exceeding a health system’s available budget. Estimates depend on local drug prices, diagnostic costs, infusion capacity, toxicity management and subsequent treatment. Extrapolating survival beyond trial follow-up introduces further uncertainty, especially when overall survival remains immature. CHEERS 2022 supports transparent reporting of these assumptions and the evaluation perspective. It does not supply a universal willingness-to-pay threshold or prove that a particular biomarker strategy is economically attractive (94).

The proposed tiers should be evaluated using explicit service outcomes: the proportion of eligible patients receiving a valid treatment-defining test, time from diagnostic sampling to treatment, the proportion unable to initiate indicated therapy, patient out-of-pocket costs and access to urgent toxicity assessment. These outcomes should be stratified by geography, center type and socioeconomic circumstances where data collection is appropriate. A research assay should be considered feasible only if its additional information can be delivered within the relevant decision window without reducing access to established care. This provides a testable implementation agenda rather than assuming that adoption of more complex platforms necessarily improves equity.

Recent implementation data make the equity discussion more concrete. A 2026 Epic Cosmos analysis included 6,689 patients receiving neoadjuvant treatment for TNBC and reported increasing pembrolizumab adoption between 2021 and 2024. It found lower use in areas with greater socioeconomic deprivation, but no significant overall difference by race or ethnicity. A separate Flatiron-based metastatic study reported variation in documented molecular testing and did not find statistically significant survival differences across its sociodemographic groups. These results support measurement of specific access gaps while cautioning against a universal assertion that every racial group experiences the same disparity in every dataset. Neither observational study can establish that the absence of a statistically significant difference proves equity (95, 96).

Diagnostic implementation has its own constraints. An Italian survey of pathology departments examined real-world SP142 testing practice, illustrating that availability and scoring processes must be assessed locally. The relevant system-level questions include specimen transport, test turnaround, training and access to the assay linked to the proposed regimen. Improvements in testing alone should not be presented as an established survival intervention unless the pathway from test completion to effective treatment is evaluated (97).

Evidence linking routine PD-L1 testing performance to ICI access and outcomes remains limited across non-Western TNBC populations and health systems. Existing studies, including Chinese investigations of PD-L1 assay concordance, demonstrate that regional evidence is available, but do not establish transportability across assays, treatment regimens or care settings. A priority is to develop multicenter routine-care cohorts that connect laboratory quality measures and testing completeness with treatment initiation, toxicity and clinical outcomes. Such studies should report local eligibility criteria, reimbursement and specimen pathways rather than interpreting geographic differences as biological differences alone. Country-specific validation is particularly important where the available assays and ICI regimens differ from those used in the pivotal trials (98).

Published economic modeling provides a useful example of the distinction between observed and projected outcomes. A United States evaluation of the KEYNOTE-522 strategy used a multistate model, trial inputs and a payer perspective to estimate lifetime cost-effectiveness. Its projected survival and quality-adjusted survival gains were model outputs, not directly observed trial outcomes. The model also preceded the later mature overall survival report. Such analyses should be reassessed when survival evidence, drug prices or treatment alternatives change, and their results should not be transferred unchanged to another health system (1, 106).

### 7.4 Special populations and the limits of transportability

Patients with autoimmune disease, frailty or substantial organ dysfunction may differ from pivotal-trial participants in both treatment risk and the ability to complete therapy. A multicenter cohort of patients with preexisting autoimmune disease documented clinical experience with ICIs across cancers, supporting individualized consideration but not a TNBC-specific safety estimate. The autoimmune diagnosis, baseline activity, immunosuppressive treatment and consequences of a flare are more informative than a single yes-or-no label. These variables should be assessed alongside the expected anticancer benefit and available alternatives, with appropriate specialist input (99).

For brain or liver metastases, the relevant evidence should describe site-specific eligibility and outcomes. Systemic response does not automatically establish intracranial activity, and a study limited to stable, previously treated brain metastases cannot resolve management of active symptomatic disease. Similarly, an organ-specific progression event may reflect a distinct biological or pharmacological environment without proving one universal resistance mechanism. Future trials should report active versus stable intracranial disease, local treatment and corticosteroid exposure, rather than subsuming these features within an overall metastatic label.

Age alone is also an incomplete description of treatment suitability. Functional status, comorbidity, medication burden, support and patient priorities influence the acceptability of prolonged combination treatment. An evidence-based discussion should avoid assuming either equal tolerability or inevitable intolerance in older adults when the relevant subgroup is small. In routine care, documenting why treatment was modified can help distinguish a necessary adaptation from an avoidable access problem. These considerations support prospective collection of patient-centered outcomes rather than automatic exclusion or indiscriminate intensification.

### 7.5 Electronic symptoms and wearable signals

Randomized electronic symptom-monitoring studies in broader oncology populations provide a stronger clinical foundation than speculative claims that wearables can predict immune toxicity. Basch and colleagues tested symptom reporting during routine cancer treatment, and the community-based PRO-TECT study evaluated electronic monitoring with patient-reported outcomes. These interventions connect patient reports to a clinical response pathway. They do not validate a smartwatch-derived algorithm for diagnosing TNBC pneumonitis, myocarditis or endocrine dysfunction. Their relevant lesson is that information becomes useful when a responsible team can interpret and act on it (100, 101).

Wearable heart-rate, activity or oxygen-saturation signals are nonspecific and can change with infection, anemia, sleep, exercise or device error. If investigated in TNBC, such systems should have a predefined intended use, alert threshold, escalation pathway and assessment of false alarms. Patients without compatible devices or reliable internet access need an equivalent route to report symptoms. Prospective evaluation should measure response time, clinically important events, workload and patient burden, not only algorithmic discrimination. **Figure 4** therefore includes patient-reported monitoring as part of implementation evaluation while retaining wearable prediction as an investigational adjunct.

**Figure 4:**
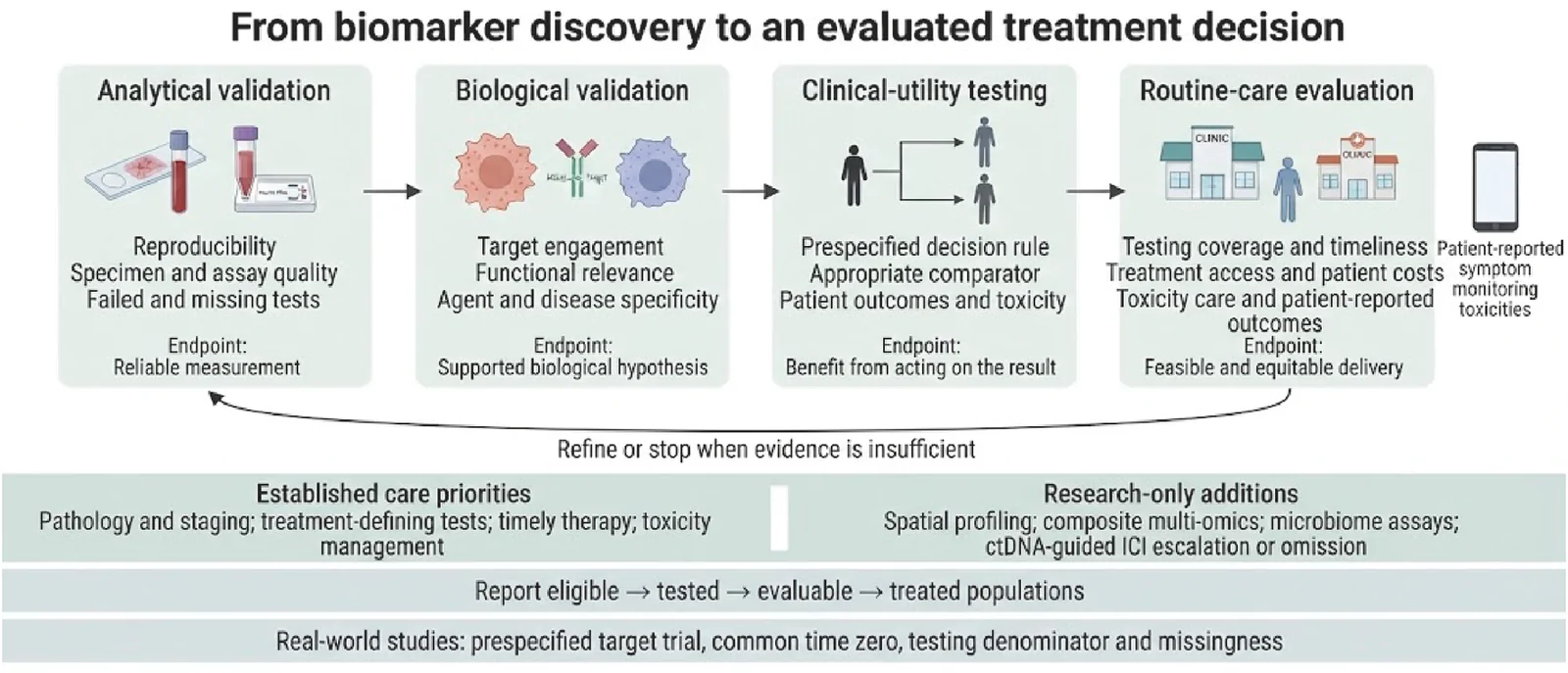
Proposed pathway from biomarker discovery to a treatment decision. The four sequential stages are analytical validation, confirmation of a biologically relevant treatment effect, prospective comparative testing of the biomarker-guided decision, and evaluation of routine-care delivery. Each stage includes an explicit endpoint: reproducibility; target engagement; clinical benefit and toxicity; and access, feasibility and patient-reported outcomes. Feedback from an unsuccessful stage returns the hypothesis for refinement. The diagram is an author-proposed research framework and contains no simulated patient data, validated clinical score or automated prescribing algorithm. Patient-reported symptom monitoring is included in implementation evaluation; wearable-based prediction remains an investigational adjunct.

### 7.6 A testable research agenda

A prospective resistance-directed study should connect four elements: a reproducible measurement, a biological hypothesis, a treatment that changes the measured process and a clinical endpoint meaningful to patients. Biomarker-positive and biomarker-negative groups should be retained when needed to test an interaction, rather than assuming predictive value from an enriched single-arm cohort. Early molecular or pathological response can inform development, but event-free survival, progression-free survival, overall survival and patient-reported outcomes remain necessary according to setting.

MORPHEUS-panBC (NCT03424005) provides an example of a phase Ib/II randomized umbrella platform evaluating multiple treatment combinations in advanced breast cancer. Its registration establishes the platform design; conclusions about TNBC efficacy require results from the relevant cohort, regimen and comparator (102).

**Figure 4** summarizes a proposed sequence for developing such evidence: validate the assay, confirm biological activity, test the treatment decision in a comparative trial, and assess delivery in routine care. These steps allow promising findings to advance while protecting established care from premature replacement. Failure at any step is informative and should be reported alongside positive findings.

An adaptive design must additionally protect interpretation when the biomarker changes during treatment. If the decision rule is updated after observing outcomes, the updated version requires a new evaluation rather than being treated as prospectively validated. A ctDNA-guided study should specify the sampling schedule and action before enrollment; a spatial-model study should fix tissue processing and computational procedures; an AI-supported trial should describe how clinicians see and use the output. Reporting frameworks can improve transparency, but the decisive evidence remains whether the resulting strategy improves outcomes compared with a credible alternative (60, 61).

Priority should be given to comparisons that resolve decisions patients already face: the contribution of postoperative treatment after a strong response, selection of a partner for an ICI, sequencing after prior ADC or perioperative immunotherapy, and intervention in molecular residual disease. Mechanistic sampling should be embedded where it can distinguish plausible explanations for success or failure. In parallel, implementation studies should measure whether the strategy can reach eligible patients without creating disproportionate diagnostic delay or toxicity burden. This connects laboratory depth with clinical relevance while preserving uncertainty where the evidence remains incomplete.

## 8 Conclusion

Biomarker-guided treatment of immune exclusion and resistance in TNBC requires a defined link between a measurement, a treatment decision and improved clinical outcomes. PD-L1 retains setting-specific clinical utility, whereas spatial profiling, ctDNA-MRD-directed decisions and multimodal signatures remain investigational. In particular, ctDNA negativity does not establish that adjuvant immunotherapy can be omitted. Positive ADC–ICI trials establish benefit for the tested regimen and population, not a universal ADC immune-priming effect or proof of immunological synergy. Mechanistic strategies must be judged against human TNBC evidence, including negative randomized comparisons and the limits of cross-disease extrapolation. Future composite models should demonstrate incremental value beyond standard clinical assessment, remain assessable when data are missing and undergo prospective clinical-utility testing. Progress will also require reliable access to established diagnostics and real-world studies that measure who is tested, treated and excluded from care.

## Supplementary material

Supplementary Table 1 ADC studies relevant to immunotherapy in TNBC.

Supplementary Table 2 Biomarker evidence and permissible interpretation.

Supplementary Table 3 Resistance directed hypotheses and clinical evidence.

## References

[1] Schmid P Cortes J Dent R McArthur H Pusztai L Kümmel S . Overall survival with pembrolizumab in early-stage triple-negative breast cancer. N Engl J Med. (2024) 391:1981–91. doi: 10.1056/nejmoa2409932 39282906

[2] Cortes J Rugo HS Cescon DW Im S-A Yusof MM Gallardo C . Pembrolizumab plus chemotherapy in advanced triple-negative breast cancer. N Engl J Med. (2022) 387:217–26. doi: 10.1056/nejmoa2202809 35857659

[3] Wang XQ Danenberg E Huang C-S Egle D Callari M Bermejo B . Spatial predictors of immunotherapy response in triple-negative breast cancer. Nature. (2023) 621:868–76. doi: 10.1038/s41586-023-06498-3 37674077 PMC10533410

[4] Lehmann BD Jovanović B Chen X Estrada MV Johnson KN Shyr Y . Refinement of triple-negative breast cancer molecular subtypes: Implications for neoadjuvant chemotherapy selection. PloS One. (2016) 11:e0157368. doi: 10.1371/journal.pone.0157368 27310713 PMC4911051

[5] Keren L Bosse M Marquez D Angoshtari R Jain S Varma S . A structured tumor-immune microenvironment in triple negative breast cancer revealed by multiplexed ion beam imaging. Cell. (2018) 174:1373–87.e19. doi: 10.1016/j.cell.2018.08.039 30193111 PMC6132072

[6] Bassez A Vos H Van Dyck L Floris G Arijs I Desmedt C . A single-cell map of intratumoral changes during anti-PD1 treatment of patients with breast cancer. Nat Med. (2021) 27:820–32. doi: 10.1038/s41591-021-01323-8 33958794

[7] Zhang Y Chen H Mo H Hu X Gao R Zhao Y . Single-cell analyses reveal key immune cell subsets associated with response to PD-L1 blockade in triple-negative breast cancer. Cancer Cell. (2021) 39:1578–93.e8. doi: 10.1016/j.ccell.2021.09.010 34653365

[8] Schmid P Cortes J Pusztai L McArthur H Kümmel S Bergh J . Pembrolizumab for early triple-negative breast cancer. N Engl J Med. (2020) 382:810–21. doi: 10.1056/nejmoa1910549 32101663

[9] Turner NC Swift C Jenkins B Kilburn L Coakley M Beaney M . Results of the c-TRAK TN trial: A clinical trial utilising ctDNA mutation tracking to detect molecular residual disease and trigger intervention in patients with moderate- and high-risk early-stage triple-negative breast cancer. Ann Oncol. (2023) 34:200–11. doi: 10.1016/j.annonc.2022.11.005 36423745

[10] Pusztai L Denkert C O’Shaughnessy J Cortes J Dent R McArthur H . Event-free survival by residual cancer burden with pembrolizumab in early-stage TNBC: Exploratory analysis from KEYNOTE-522. Ann Oncol. (2024) 35:429–36. doi: 10.1016/j.annonc.2024.02.002 38369015

[11] Mittendorf EA Assaf ZJ Harbeck N Zhang H Saji S Jung KH . Peri-operative atezolizumab in early-stage triple-negative breast cancer: Final results and ctDNA analyses from the randomized phase 3 IMpassion031 trial. Nat Med. (2025) 31:2397–404. doi: 10.1038/s41591-025-03725-4 40467898 PMC12283416

[12] Loibl S Untch M Huober J Schaser V Braun M Denkert C . Durvalumab in combination with neoadjuvant chemotherapy in early triple-negative breast cancer: Long-term analysis from the GeparNuevo trial. J Clin Oncol. (2026) 44:1580–6. doi: 10.1200/jco-25-02311 42008768

[13] Ignatiadis M Bailey A McArthur H El-Abed S de Azambuja E Metzger O . Adjuvant atezolizumab for early triple-negative breast cancer: The ALEXANDRA/IMpassion030 randomized clinical trial. JAMA. (2025) 333:1150–60. doi: 10.1001/jama.2024.26886 39883436 PMC11783246

[14] Sharma P Stecklein SR Yoder R Staley JM Schwensen K O’Dea A . Clinical and biomarker findings of neoadjuvant pembrolizumab and carboplatin plus docetaxel in triple-negative breast cancer: NeoPACT phase 2 clinical trial. JAMA Oncol. (2024) 10:227–35. doi: 10.1001/jamaoncol.2023.5033 37991778 PMC10666040

[15] Schmid P Adams S Rugo HS Schneeweiss A Barrios CH Iwata H . Atezolizumab and nab-paclitaxel in advanced triple-negative breast cancer. N Engl J Med. (2018) 379:2108–21. doi: 10.1056/nejmoa1809615 30345906

[16] Rugo HS Loi S Adams S Schmid P Schneeweiss A Barrios CH . PD-L1 immunohistochemistry assay comparison in atezolizumab plus nab-paclitaxel-treated advanced triple-negative breast cancer. J Natl Cancer Institute. (2021) 113:1733–43. doi: 10.1093/jnci/djab108 34097070 PMC8634452

[17] Miles D Gligorov J André F Cameron D Schneeweiss A Barrios C . Primary results from IMpassion131, a double-blind, placebo-controlled, randomised phase III trial of first-line paclitaxel with or without atezolizumab for unresectable locally advanced/metastatic triple-negative breast cancer. Ann Oncol. (2021) 32:994–1004. doi: 10.1016/j.annonc.2021.05.801 34219000

[18] U.S. Food and Drug Administration . CDER drug and biologic accelerated approvals based on a surrogate endpoint (2026). Available online at: https://www.fda.gov/media/151146/download (Accessed September 8, 2026).

[19] Emens LA Cruz C Eder JP Braiteh F Chung C Tolaney SM . Long-term clinical outcomes and biomarker analyses of atezolizumab therapy for patients with metastatic triple-negative breast cancer: A phase 1 study. JAMA Oncol. (2019) 5:74–82. doi: 10.1001/jamaoncol.2018.4224 30242306 PMC6439773

[20] Winer EP Lipatov O Im S-A Goncalves A Muñoz-Couselo E Lee KS . Pembrolizumab versus investigator-choice chemotherapy for metastatic triple-negative breast cancer (KEYNOTE-119): A randomised, open-label, phase 3 trial. Lancet Oncol. (2021) 22:499–511. doi: 10.1016/s1470-2045(20)30754-3 33676601

[21] Cortes J Cescon DW Rugo HS Nowecki Z Im S-A Yusof MM . Pembrolizumab plus chemotherapy versus placebo plus chemotherapy for previously untreated locally recurrent inoperable or metastatic triple-negative breast cancer (KEYNOTE-355): A randomised, placebo-controlled, double-blind, phase 3 clinical trial. Lancet. (2020) 396:1817–28. doi: 10.1016/s0140-6736(20)32531-9 33278935

[22] Tolaney SM de Azambuja E Kalinsky K Loi S Kim S-B Yam C . Sacituzumab govitecan plus pembrolizumab for advanced triple-negative breast cancer. N Engl J Med. (2026) 394:354–66. doi: 10.1056/nejmoa2508959 41564397

[23] U.S. Food and Drug Administration . FDA approves sacituzumab govitecan-hziy as monotherapy and in combination with pembrolizumab for first-line treatment of triple-negative breast cancer (2026). Available online at: https://www.fda.gov/drugs/resources-information-approved-drugs/fda-approves-sacituzumab-govitecan-hziy-monotherapy-and-combination-pembrolizumab-first-line (Accessed September 8, 2026).

[24] Cortés J Punie K Barrios C Hurvitz SA Schneeweiss A Sohn J . Sacituzumab govitecan in untreated, advanced triple-negative breast cancer. N Engl J Med. (2025) 393:1912–25. doi: 10.1056/nejmoa2511734 41124233

[25] GBG Forschungs GmbH . Sacituzumab govitecan in primary HER2-negative breast cancer (NCT04595565). In: Clinicaltrials.Gov. Bethesda, MD: U.S. National Library of Medicine (ClinicalTrials.gov) (2026). Available online at: https://clinicaltrials.gov/study/NCT04595565 (Accessed September 8, 2026).

[26] Iwata TN Ishii C Ishida S Ogitani Y Wada T Agatsuma T . A HER2-targeting antibody-drug conjugate, trastuzumab deruxtecan (DS-8201a), enhances antitumor immunity in a mouse model. Mol Cancer Ther. (2018) 17:1494–503. doi: 10.1158/1535-7163.mct-17-0749 29703841

[27] Galluzzi L Vitale I Warren S Adjemian S Agostinis P Martinez AB . Consensus guidelines for the definition, detection and interpretation of immunogenic cell death. J Immunother Cancer. (2020) 8:e000337. doi: 10.1136/jitc-2019-000337 32209603 PMC7064135

[28] Sharkey RM McBride WJ Cardillo TM Govindan SV Wang Y Rossi EA . Enhanced delivery of SN-38 to human tumor xenografts with an anti-trop-2-SN-38 antibody conjugate (Sacituzumab govitecan). Clin Cancer Res. (2015) 21:5131–8. doi: 10.1158/1078-0432.CCR-15-0670 26106073

[29] Okajima D Yasuda S Maejima T Karibe T Sakurai K Aida T . Datopotamab deruxtecan, a novel TROP2-directed antibody-drug conjugate, demonstrates potent antitumor activity by efficient drug delivery to tumor cells. Mol Cancer Ther. (2021) 20:2329–40. doi: 10.1158/1535-7163.MCT-21-0206 34413126 PMC9398094

[30] Schmid P Oliveira M O’Shaughnessy J Cristofanilli M Graff SL Im S-A . TROPION-Breast05: A randomized phase III study of Dato-DXd with or without durvalumab versus chemotherapy plus pembrolizumab in patients with PD-L1-high locally recurrent inoperable or metastatic triple-negative breast cancer. Ther Adv Med Oncol. (2025) 17:17588359251327992. doi: 10.1177/17588359251327992 40297626 PMC12035291

[31] Modi S Jacot W Yamashita T Sohn J Vidal M Tokunaga E . Trastuzumab deruxtecan in previously treated HER2-low advanced breast cancer. N Engl J Med. (2022) 387:9–20. doi: 10.1056/nejmoa2203690 35665782 PMC10561652

[32] Daiichi Sankyo . DS8201a and pembrolizumab in participants with locally advanced/metastatic breast or non-small cell lung cancer (NCT04042701). In: Clinicaltrials.Gov. Bethesda, MD: U.S. National Library of Medicine (ClinicalTrials.gov) (2025). Available online at: https://clinicaltrials.gov/study/NCT04042701 (Accessed September 8, 2026).

[33] Rugo HS Torres AA Arkenau H-T Kipps E Italiano A Blanco AC . Trastuzumab deruxtecan with pembrolizumab in previously treated HER2-expressing advanced or metastatic breast cancer: Interim analyses of the breast cohorts from the open-label, multicenter, phase 1b study DS8201-A-U106 [Conference abstract. Clin Cancer Res. (2025) 31:P5-07-29. doi: 10.1158/1557-3265.sabcs24-p5-07-2936230740

[34] Schmid P Im S-A Nowecki Z Wysocki PJ Jassem J Jung KH . First-line durvalumab in combination with trastuzumab deruxtecan in women with locally advanced unresectable or metastatic, hormone-receptor-negative, HER2-low breast cancer: Multicenter, open-label, phase 1b/2 BEGONIA platform trial. Nat Cancer. (2026) 7:983–92. doi: 10.1038/s43018-026-01181-8 42259988 PMC13309285

[35] Bardia A Tolaney SM Punie K Loirat D Oliveira M Kalinsky K . Biomarker analyses in the phase III ASCENT study of sacituzumab govitecan versus chemotherapy in patients with metastatic triple-negative breast cancer. Ann Oncol. (2021) 32:1148–56. doi: 10.1016/j.annonc.2021.06.002 34116144

[36] Coates JT Sun S Leshchiner I Thimmiah N Martin EE McLoughlin D . Parallel genomic alterations of antigen and payload targets mediate polyclonal acquired clinical resistance to sacituzumab govitecan in triple-negative breast cancer. Cancer Discov. (2021) 11:2436–45. doi: 10.1158/2159-8290.cd-21-0702 34404686 PMC8495771

[37] Poumeaud F Morisseau M Cabel L Gonçalves A Rivier C Trédan O . Efficacy of administration sequence: Sacituzumab govitecan and trastuzumab deruxtecan in HER2-low metastatic breast cancer. Br J Cancer. (2024) 131:702–8. doi: 10.1038/s41416-024-02766-9 38918555 PMC11333747

[38] Leon-Ferre RA Jonas SF Salgado R Loi S de Jong V Carter JM . Tumor-infiltrating lymphocytes in triple-negative breast cancer. JAMA. (2024) 331:1135–44. doi: 10.1001/jama.2024.3056 PMC10988354 38563834

[39] Helmink BA Reddy SM Gao J Zhang S Basar R Thakur R . B cells and tertiary lymphoid structures promote immunotherapy response. Nature. (2020) 577:549–55. doi: 10.1038/s41586-019-1922-8 31942075 PMC8762581

[40] Salgado R Denkert C Demaria S Sirtaine N Klauschen F Pruneri G . The evaluation of tumor-infiltrating lymphocytes (TILs) in breast cancer: Recommendations by an International TILs Working Group 2014. Ann Oncol. (2015) 26:259–71. doi: 10.1093/annonc/mdu450 25214542 PMC6267863

[41] Loi S Drubay D Adams S Pruneri G Francis PA Lacroix-Triki M . Tumor-infiltrating lymphocytes and prognosis: A pooled individual patient analysis of early-stage triple-negative breast cancers. J Clin Oncol. (2019) 37:559–69. doi: 10.1200/jco.18.01010 30650045 PMC7010425

[42] de Jong VMT Wang Y Ter Hoeve ND Opdam M Stathonikos N Jóźwiak K . Prognostic value of stromal tumor-infiltrating lymphocytes in young, node-negative, triple-negative breast cancer patients who did not receive (neo)adjuvant systemic therapy. J Clin Oncol. (2022) 40:2361–74. doi: 10.1200/jco.21.01536 35353548 PMC9287283

[43] Denkert C von Minckwitz G Darb-Esfahani S Lederer B Heppner BI Weber KE . Tumour-infiltrating lymphocytes and prognosis in different subtypes of breast cancer: A pooled analysis of 3771 patients treated with neoadjuvant therapy. Lancet Oncol. (2018) 19:40–50. doi: 10.1016/s1470-2045(17)30904-x 29233559

[44] Robson M Im S-A Senkus E Xu B Domchek SM Masuda N . Olaparib for metastatic breast cancer in patients with a germline BRCA mutation. N Engl J Med. (2017) 377:523–33. doi: 10.1056/nejmoa1706450 28578601

[45] Tutt ANJ Garber JE Kaufman B Viale G Fumagalli D Rastogi P . Adjuvant olaparib for patients with BRCA1- or BRCA2-mutated breast cancer. N Engl J Med. (2021) 384:2394–405. doi: 10.1056/nejmoa2105215 34081848 PMC9126186

[46] Tan TJ Sammons S Im Y-H She L Mundy K Bigelow R . Phase II DORA study of olaparib with or without durvalumab as a chemotherapy-free maintenance strategy in platinum-pretreated advanced triple-negative breast cancer. Clin Cancer Res. (2024) 30:1240–7. doi: 10.1158/1078-0432.ccr-23-2513 38236575 PMC10982642

[47] U.S. Food and Drug Administration . FDA approves pembrolizumab for adults and children with TMB-H solid tumors (2020). Available online at: https://www.fda.gov/drugs/drug-approvals-and-databases/fda-approves-pembrolizumab-adults-and-children-tmb-h-solid-tumors (Accessed September 9, 2026).

[48] U.S. Food and Drug Administration . List of FDA-authorized companion diagnostic devices (*in vitro* and imaging tools). Available online at: https://www.fda.gov/medical-devices/in-vitro-diagnostics/list-fda-authorized-companion-diagnostic-devices-in-vitro-and-imaging-tools (Accessed September 9, 2026).

[49] Simon RM Paik S Hayes DF . Use of archived specimens in evaluation of prognostic and predictive biomarkers. J Natl Cancer Institute. (2009) 101:1446–52. doi: 10.1093/jnci/djp335 19815849 PMC2782246

[50] Simon R Radmacher MD Dobbin K McShane LM . Pitfalls in the use of DNA microarray data for diagnostic and prognostic classification. J Natl Cancer Institute. (2003) 95:14–8. doi: 10.1093/jnci/95.1.14 12509396

[51] Pascual J Attard G Bidard FC Curigliano G De Mattos-Arruda L Diehn M . ESMO recommendations on the use of circulating tumour DNA assays for patients with cancer: a report from the ESMO Precision Medicine Working Group. Ann Oncol Off J Eur Soc For Med Oncol. (2022) 33:750–68. doi: 10.1016/j.annonc.2022.05.520 35809752

[52] Coombes RC Page K Salari R Hastings RK Armstrong A Ahmed S . Personalized detection of circulating tumor DNA antedates breast cancer metastatic recurrence. Clin Cancer Res. (2019) 25:4255–63. doi: 10.1158/1078-0432.ccr-18-3663 30992300

[53] Radovich M Jiang G Hancock BA Chitambar C Nanda R Falkson C . Association of circulating tumor DNA and circulating tumor cells after neoadjuvant chemotherapy with disease recurrence in patients with triple-negative breast cancer: Preplanned secondary analysis of the BRE12-158 randomized clinical trial. JAMA Oncol. (2020) 6:1410–5. doi: 10.1001/jamaoncol.2020.2295 32644110 PMC7349081

[54] Shaw JA Page K Wren E de Bruin EC Kalashnikova E Hastings R . Serial postoperative circulating tumor DNA assessment has strong prognostic value during long-term follow-up in patients with breast cancer. JCO Precis Oncol. (2024) 8:e2300456. doi: 10.1200/po.23.00456 38691816 PMC11161241

[55] Hu Y Ulrich BC Supplee J Kuang Y Lizotte PH Feeney NB . False-positive plasma genotyping due to clonal hematopoiesis. Clin Cancer Res. (2018) 24:4437–43. doi: 10.1158/1078-0432.ccr-18-0143 29567812

[56] Schneider BP Jiang G Ballinger TJ Shen F Chitambar C Nanda R . BRE12-158: A postneoadjuvant, randomized phase II trial of personalized therapy versus treatment of physician’s choice for patients with residual triple-negative breast cancer. J Clin Oncol. (2022) 40:345–55. doi: 10.1200/jco.21.01657 34910554

[57] Davar D Dzutsev AK McCulloch JA Rodrigues RR Chauvin J-M Morrison RM . Fecal microbiota transplant overcomes resistance to anti-PD-1 therapy in melanoma patients. Science. (2021) 371:595–602. doi: 10.1126/science.abf3363 33542131 PMC8097968

[58] Routy B Le Chatelier E Derosa L Duong CPM Alou MT Daillère R . Gut microbiome influences efficacy of PD-1-based immunotherapy against epithelial tumors. Science. (2018) 359:91–7. doi: 10.1126/science.aan3706 29097494

[59] Spencer CN McQuade JL Gopalakrishnan V McCulloch JA Vetizou M Cogdill AP . Dietary fiber and probiotics influence the gut microbiome and melanoma immunotherapy response. Science. (2021) 374:1632–40. doi: 10.1126/science.aaz7015 34941392 PMC8970537

[60] Collins GS Moons KGM Dhiman P Riley RD Beam AL Van Calster B . TRIPOD+AI statement: Updated guidance for reporting clinical prediction models that use regression or machine learning methods. BMJ. (2024) 385:e078378. doi: 10.1136/bmj-2023-078378 38626948 PMC11019967

[61] Liu X Cruz Rivera S Moher D Calvert MJ Denniston AK SPIRIT-AI and CONSORT-AI Working Group . Reporting guidelines for clinical trial reports for interventions involving artificial intelligence: The CONSORT-AI extension. Nat Med. (2020) 26:1364–74. doi: 10.1038/s41591-020-1034-x 32908283 PMC7598943

[62] Luke JJ Bao R Sweis RF Spranger S Gajewski TF . WNT/β-catenin pathway activation correlates with immune exclusion across human cancers. Clin Cancer Res. (2019) 25:3074–83. doi: 10.1158/1078-0432.ccr-18-1942 30635339 PMC6522301

[63] Zaretsky JM Garcia-Diaz A Shin DS Escuin-Ordinas H Hugo W Hu-Lieskovan S . Mutations associated with acquired resistance to PD-1 blockade in melanoma. N Engl J Med. (2016) 375:819–29. doi: 10.1056/nejmoa1604958 27433843 PMC5007206

[64] Costa A Kieffer Y Scholer-Dahirel A Pelon F Bourachot B Cardon M . Fibroblast heterogeneity and immunosuppressive environment in human breast cancer. Cancer Cell. (2018) 33:463–79.e10. doi: 10.1016/j.ccell.2018.01.011 29455927

[65] Mariathasan S Turley SJ Nickles D Castiglioni A Yuen K Wang Y . TGFβ attenuates tumour response to PD-L1 blockade by contributing to exclusion of T cells. Nature. (2018) 554:544–8. doi: 10.1038/nature25501 29443960 PMC6028240

[66] Spranger S Bao R Gajewski TF . Melanoma-intrinsic β-catenin signalling prevents anti-tumour immunity. Nature. (2015) 523:231–5. doi: 10.1038/nature14404 25970248

[67] Roussos Torres ET Ho WJ Danilova L Tandurella JA Leatherman J Rafie C . Entinostat, nivolumab and ipilimumab for women with advanced HER2-negative breast cancer: A phase Ib trial. Nat Cancer. (2024) 5:866–79. doi: 10.1038/s43018-024-00729-w 38355777 PMC11552660

[68] Cheng J Ge T Zhu X Wang J Zeng Y Mu W . Preclinical development and evaluation of nanobody-based CD70-specific CAR T cells for the treatment of acute myeloid leukemia. Cancer Immunol Immunother. (2023) 72:2331–46. doi: 10.1007/s00262-023-03422-6 36932256 PMC10264288

[69] Buisseret L Loirat D Aftimos P Maurer C Punie K Debien V . Paclitaxel plus carboplatin and durvalumab with or without oleclumab for women with previously untreated locally advanced or metastatic triple-negative breast cancer: The randomized SYNERGY phase I/II trial. Nat Commun. (2023) 14:7018. doi: 10.1038/s41467-023-42744-y 37919269 PMC10622534

[70] Dent RA Kim S-B Oliveira M Barrios C O’Shaughnessy J Isakoff SJ . Ipatasertib plus paclitaxel for patients with PIK3CA/AKT1/PTEN-altered locally advanced unresectable or metastatic triple-negative breast cancer in the IPATunity130 phase III trial. Clin Cancer Res. (2024) 30:4329–38. doi: 10.1158/1078-0432.ccr-24-0465 39058425 PMC11443247

[71] Stagg J Divisekera U McLaughlin N Sharkey J Pommey S Denoyer D . Anti-CD73 antibody therapy inhibits breast tumor growth and metastasis. PNAS. (2010) 107:1547–52. doi: 10.1073/pnas.0908801107 20080644 PMC2824381

[72] Loi S Pommey S Haibe-Kains B Beavis PA Darcy PK Smyth MJ . CD73 promotes anthracycline resistance and poor prognosis in triple negative breast cancer. PNAS. (2013) 110:11091–6. doi: 10.1073/pnas.1222251110 23776241 PMC3704029

[73] Song X Zhou X Qin Y Yang J Wang Y Sun Z . Emodin inhibits epithelial-mesenchymal transition and metastasis of triple negative breast cancer via antagonism of CC-chemokine ligand 5 secreted from adipocytes. Int J Mol Med. (2018) 42:579–88. doi: 10.3892/ijmm.2018.3638 29693154

[74] Qiu Y Chen A Yu R Llevenes P Seen M Ko NY . Insulin resistance increases TNBC aggressiveness and brain metastasis via adipocyte-derived exosomes. Mol Cancer Res. (2025) 23:567–78. doi: 10.1158/1541-7786.mcr-24-0494 40047645 PMC12133429

[75] Pantelidou C Sonzogni O De Oliveria Taveira M Mehta AK Kothari A Wang D . PARP inhibitor efficacy depends on CD8+ T-cell recruitment via intratumoral STING pathway activation in BRCA-deficient models of triple-negative breast cancer. Cancer Discov. (2019) 9:722–37. doi: 10.1158/2159-8290.cd-18-1218 31015319 PMC6548644

[76] Vinayak S Tolaney SM Schwartzberg L Mita M McCann G Tan AR . Open-label clinical trial of niraparib combined with pembrolizumab for treatment of advanced or metastatic triple-negative breast cancer. JAMA Oncol. (2019) 5:1132–40. doi: 10.1001/jamaoncol.2019.1029 31194225 PMC6567845

[77] Domchek SM Postel-Vinay S Im S-A Park YH Delord J-P Italiano A . Olaparib and durvalumab in patients with germline BRCA-mutated metastatic breast cancer (MEDIOLA): An open-label, multicentre, phase 1/2, basket study. Lancet Oncol. (2020) 21:1155–64. doi: 10.1016/s1470-2045(20)30324-7 32771088

[78] Voorwerk L Slagter M Horlings HM Sikorska K van de Vijver KK de Maaker M . Immune induction strategies in metastatic triple-negative breast cancer to enhance the sensitivity to PD-1 blockade: The TONIC trial. Nat Med. (2019) 25:920–8. doi: 10.1038/s41591-019-0432-4 31086347

[79] Ho AY Barker CA Arnold BB Powell SN Hu ZI Gucalp A . A phase 2 clinical trial assessing the efficacy and safety of pembrolizumab and radiotherapy in patients with metastatic triple-negative breast cancer. Cancer. (2020) 126:850–60. doi: 10.1002/cncr.32599 31747077

[80] Soliman H Hogue D Han H Mooney B Costa R Lee MC . Oncolytic T-VEC virotherapy plus neoadjuvant chemotherapy in nonmetastatic triple-negative breast cancer: A phase 2 trial. Nat Med. (2023) 29:450–7. doi: 10.1038/s41591-023-02210-0 36759673

[81] Pascual T Vidal M Cejalvo JM Vega E Sanfeliu E Villacampa G . Talimogene laherparepvec and atezolizumab in HER2-negative breast cancer following neoadjuvant chemotherapy: A window-of-opportunity phase II trial (SOLTI-1503 PROMETEO). Nat Commun. (2026) 17:2817. doi: 10.1038/s41467-026-69222-5 41698899 PMC13021909

[82] Nederlof I Isaeva OI de Graaf M Gielen RCAM Bakker NAM Rolfes AL . Neoadjuvant nivolumab or nivolumab plus ipilimumab in early-stage triple-negative breast cancer: A phase 2 adaptive trial. Nat Med. (2024) 30:3223–35. doi: 10.1038/s41591-024-03249-3 39284953 PMC11564107

[83] Cheng J Zhu L Wu J Zeng Y Mao X Ding S . Efficacy and safety of autologous CD5-KO anti-CD5 CAR-T cells in relapsed/refractory CD5+ hematological malignancies. Cell Rep Med. (2026) 7:102584. doi: 10.1016/j.xcrm.2026.102584 41633358 PMC12923949

[84] Zacharakis N Chinnasamy H Black M Xu H Lu Y-C Zheng Z . Immune recognition of somatic mutations leading to complete durable regression in metastatic breast cancer. Nat Med. (2018) 24:724–30. doi: 10.1038/s41591-018-0040-8 29867227 PMC6348479

[85] Zhang X Goedegebuure SP Chen MY Mishra R Zhang F Yu YY . Neoantigen DNA vaccines are safe, feasible, and induce neoantigen-specific immune responses in triple-negative breast cancer patients. Genome Med. (2024) 16:131. doi: 10.1186/s13073-024-01388-3 39538331 PMC11562513

[86] Sahin U Schmidt M Derhovanessian E Cortini A Vogler I Omokoko T . Individualized mRNA vaccines evoke durable T cell immunity in adjuvant TNBC. Nature. (2026) 651:1088–96. doi: 10.1038/s41586-025-10004-2 41708868 PMC13017525

[87] Masuda N Lee S-J Ohtani S Im Y-H Lee E-S Yokota I . Adjuvant capecitabine for breast cancer after preoperative chemotherapy. N Engl J Med. (2017) 376:2147–59. doi: 10.1056/nejmoa1612645 28564564

[88] Schneider BJ Naidoo J Santomasso BD Lacchetti C Adkins S Anadkat M . Management of immune-related adverse events in patients treated with immune checkpoint inhibitor therapy: ASCO guideline update. J Clin Oncol. (2021) 39:4073–126. doi: 10.1200/jco.21.01440 34724392

[89] Brahmer JR Abu-Sbeih H Ascierto PA Brufsky J Cappelli LC Cortazar FB . Society for Immunotherapy of Cancer (SITC) clinical practice guideline on immune checkpoint inhibitor-related adverse events. J Immunother Cancer. (2021) 9:e002435. doi: 10.1136/jitc-2021-002435 34172516 PMC8237720

[90] Ricciuti B Dahlberg SE Adeni A Sholl LM Nishino M Awad MM . Immune checkpoint inhibitor outcomes for patients with non-small-cell lung cancer receiving baseline corticosteroids for palliative versus nonpalliative indications. J Clin Oncol. (2019) 37:1927–34. doi: 10.1200/jco.19.00189 31206316

[91] Loibl S Loirat D Tolaney SM Punie K Oliveira M Rugo HS . Health-related quality of life in the phase III ASCENT trial of sacituzumab govitecan versus standard chemotherapy in metastatic triple-negative breast cancer. Eur J Cancer. (2023) 178:23–33. doi: 10.1016/j.ejca.2022.10.003 36379186 PMC11195534

[92] Connors C Valente SA ElSherif A Escobar P Chichura A Kopicky L . Real-world outcomes with the KEYNOTE-522 regimen in early-stage triple-negative breast cancer. Ann Surg Oncol. (2025) 32:912–21. doi: 10.1245/s10434-024-16390-7 39436619 PMC11843215

[93] Hernán MA Robins JM . Using big data to emulate a target trial when a randomized trial is not available. Am J Epidemiol. (2016) 183:758–64. doi: 10.1093/aje/kwv254 26994063 PMC4832051

[94] Husereau D Drummond M Augustovski F de Bekker-Grob E Briggs AH Carswell C . Consolidated health economic evaluation reporting standards 2022 (CHEERS 2022) statement: Updated reporting guidance for health economic evaluations. Clin Ther. (2022) 44:158–68. doi: 10.1016/j.clinthera.2022.01.011 35168801

[95] Li E Spierling Bagsic SR Belasco RF Nguyen CH Ali S Liloia N . Trends in neoadjuvant pembrolizumab use in triple-negative breast cancer. Am J Clin Oncol. (2026). doi: 10.1097/coc.0000000000001305 41734452

[96] Tolaney SM Spring L Abdou YG Rehnquist MK Hogea C Estrin A . Understanding first-line treatment patterns and survival outcomes across sociodemographic groups of women with metastatic triple-negative breast cancer in the United States: A real-world study. ESMO Open. (2025) 10:105841. doi: 10.1016/j.esmoop.2025.105841 41061347 PMC12538425

[97] Cerbelli B Cirillo A Pomati G Pernazza A Ascione A Pisegna S . PD-L1 testing in metastatic triple negative breast cancer: Results of an Italian survey. Tumori J. (2024) 110:44–8. doi: 10.1177/03008916231196781 37726962

[98] Chen C Ma X Li Y Ma J Yang W Shui R . Concordance of PD-L1 expression in triple-negative breast cancers in Chinese patients: A retrospective and pathologist-based study. Pathol Res Pract. (2022) 238:154137. doi: 10.1016/j.prp.2022.154137 36152566

[99] Tison A Quéré G Misery L Funck-Brentano E Danlos F-X Routier E . Safety and efficacy of immune checkpoint inhibitors in patients with cancer and preexisting autoimmune disease: A nationwide, multicenter cohort study. Arthritis Rheumatol. (2019) 71:2100–11. doi: 10.1002/art.41068 31379105

[100] Basch E Deal AM Kris MG Scher HI Hudis CA Sabbatini P . Symptom monitoring with patient-reported outcomes during routine cancer treatment: A randomized controlled trial. J Clin Oncol. (2016) 34:557–65. doi: 10.1200/jco.2015.63.0830 26644527 PMC4872028

[101] Basch E Schrag D Henson S Jansen J Ginos B Stover AM . Effect of electronic symptom monitoring on patient-reported outcomes among patients with metastatic cancer: A randomized clinical trial. JAMA. (2022) 327:2413–22. doi: 10.1001/jama.2022.9265 35661856 PMC9168923

[102] Hoffmann-La Roche . A Study Evaluating the Efficacy and Safety of Multiple Treatment Combinations in Patients With Metastatic or Locally Advanced Breast Cancer (NCT03424005). Bethesda, MD: U.S. National Library of Medicine, National Center for Biotechnology Information (NCBI) (2026). Available online at: https://clinicaltrials.gov/study/NCT03424005 (Accessed September 8, 2026).

[103] Bardia A Hurvitz SA Tolaney SM Loirat D Punie K Oliveira M . Sacituzumab govitecan in metastatic triple-negative breast cancer. N Engl J Med. (2021) 384:1529–41. doi: 10.1056/nejmoa2028485 33882206

[104] Schmid P Wang H-C Lynce F Asselah J Jung KH Ma CX . Datopotamab deruxtecan (Dato-DXd) in combination with durvalumab as first-line treatment for unresectable locally advanced or metastatic triple-negative breast cancer: Results from arms 7 and 8 of the phase Ib/II BEGONIA study. Ann Oncol. (2026) 37:1081–92. doi: 10.1016/j.annonc.2026.05.693 42167437

[105] Hodgson D Lai Z Dearden S Barrett JC Harrington EA Timms K . Analysis of mutation status and homologous recombination deficiency in tumors of patients with germline BRCA1 or BRCA2 mutations and metastatic breast cancer: OlympiAD. Ann Oncol. (2021) 32:1582–9. doi: 10.1016/j.annonc.2021.08.2154 34500047

[106] Huang M Fasching PA Haiderali A Xue W Yang C Pan W . Cost-effectiveness of neoadjuvant pembrolizumab plus chemotherapy followed by adjuvant single-agent pembrolizumab for high-risk early-stage triple-negative breast cancer in the United States. Adv Ther. (2023) 40:1153–70. doi: 10.1007/s12325-022-02365-1 36648737 PMC9988745

