# Supplementary material for: Precision immuno-oncology in triple-negative breast cancer: from PD-L1 to multimodal biomarkers, ADC–ICI combinations, and global real-world integration
Source: Front Immunol. 2026 Oct 12;17:1911879. doi: 10.3389/fimmu.2026.1911879 (PMC13696950; doi:10.3389/fimmu.2026.1911879)
Supplement: Supplementary file 1 [file Table1.docx]

Supplementary Table 1 ADC studies relevant to immunotherapy in TNBC

| **Study and population** | **Regimen and design** | **Evidence supported** | **Limits on inference and safety** | **Refs.** |
| --- | --- | --- | --- | --- |
| ASCENT-04 / KEYNOTE-D19 First-line PD-L1-positive advanced TNBC | Phase III; SG + pembrolizumab versus chemotherapy + pembrolizumab; 443 patients | PFS improved; United States first-line combination approval in June 2026 for CPS ≥10 | Both arms contain pembrolizumab; no SG-alone arm. Does not isolate added pembrolizumab benefit or distinguish cytotoxic activity from an immune interaction. Does not establish immunological synergy or benefit after progression on prior ICI. OS immature in the primary report. | [22, 23] |
| ASCENT-03 ICI-ineligible advanced TNBC | Phase III; SG monotherapy versus chemotherapy | Direct evidence for first-line SG in its eligible population | Not an ADC-ICI trial; do not transfer results to SG + pembrolizumab | [24] |
| SASCIA Post-neoadjuvant residual HER2-negative breast cancer | Phase III; SG versus treatment of physician's choice | Registry supports post-neoadjuvant design | Not a neoadjuvant pCR study or an SG-ICI comparison; no efficacy result inferred from registration | [25] |
| MORPHEUS-panBC Locally advanced/metastatic breast cancer | Phase Ib/II platform; includes SG + atezolizumab investigation | Identifies early-phase combination development | Cohort-specific eligibility and comparator required; no unsupported TNBC response estimate assigned | [102] |
| BEGONIA arms 7 and 8 First-line advanced TNBC; arm 7 irrespective of PD-L1, arm 8 PD-L1-high | Phase Ib/II; Dato-DXd + durvalumab | Published combination activity and safety evidence | Exploratory cohorts; no randomized estimate of added durvalumab benefit. No cross-trial ranking against SG combinations. Toxicity must be assessed for the actual cohort and regimen. | [104] |
| TROPION-Breast05 PD-L1-high advanced TNBC | Phase III design; Dato-DXd ± durvalumab versus chemotherapy + pembrolizumab; monotherapy arm in selected countries | Design addresses clinically relevant comparative question | Protocol report is not an efficacy result; do not combine with all TROPION studies as one phase | [30] |
| DS8201-A-U106 Previously treated HER2-expressing advanced breast cancer cohorts | Phase Ib; T-DXd + pembrolizumab; NCT04042701 | Registry and conference interim report identify the combination | Mixed HER2/HR populations; no TNBC-specific benefit inferred; overlapping pulmonary toxicity requires assessment | [32, 33] |
| BEGONIA arm 6 HR-negative HER2-low advanced breast cancer | Phase Ib/II; T-DXd + durvalumab | 58 participants; ORR 62.1%; protocol response objective not met | Response objective not met despite the observed ORR. Distinct from T-DXd + pembrolizumab; no randomized estimate of the ICI contribution or proof of immune synergy. | [34] |
| DESTINY-Breast04 Previously treated HER2-low advanced breast cancer | Phase III; T-DXd monotherapy versus chemotherapy | Randomized monotherapy evidence; smaller HR-negative subgroup | Not an ICI trial; ILD/pneumonitis rates belong to this regimen and population | [31] |
| ASCENT Previously treated metastatic TNBC | Phase III; SG versus physician-choice chemotherapy | Randomized monotherapy survival evidence | Cannot be relabeled as evidence for an SG–ICI combination | [103] |

Clinical activity or comparative regimen benefit does not by itself establish immunological synergy. Proposed immune effects are ADC-, model- and setting-specific; findings must not be transferred between SG, Dato-DXd and T-DXd, checkpoint partners or prior-ICI populations without supporting evidence. Bystander killing is distinct from immune antigen spreading.

Registry and protocol entries identify the design described by the cited source; they are not efficacy results. Numerical results refer to the cited reports, not necessarily a later data cutoff. Safety estimates belong to the actual regimen and population.

Abbreviations: ADC, antibody–drug conjugate; SG, sacituzumab govitecan; Dato-DXd, datopotamab deruxtecan; T-DXd, trastuzumab deruxtecan; ICI, immune checkpoint inhibitor; TNBC, triple-negative breast cancer; CPS, combined positive score; PFS, progression-free survival; OS, overall survival; HR, hormone receptor; ORR, objective response rate; ILD, interstitial lung disease; pCR, pathological complete response.

# References for this table

[22] Tolaney SM, et al. Sacituzumab Govitecan plus Pembrolizumab for Advanced Triple-Negative Breast Cancer. N Engl J Med. 2026;394:354–366. https://doi.org/10.1056/NEJMoa2508959

[23] US Food and Drug Administration. FDA approves sacituzumab govitecan-hziy as monotherapy and in combination with pembrolizumab for first-line treatment of triple-negative breast cancer. 24 June 2026. Accessed 8 September 2026. https://www.fda.gov/drugs/resources-information-approved-drugs/fda-approves-sacituzumab-govitecan-hziy-monotherapy-and-combination-pembrolizumab-first-line

[24] Cortés J, et al. Sacituzumab Govitecan in Untreated, Advanced Triple-Negative Breast Cancer. N Engl J Med. 2025;393:1912–1925. https://doi.org/10.1056/NEJMoa2511734

[25] ClinicalTrials.gov. Sacituzumab Govitecan in Primary HER2-negative Breast Cancer (SASCIA). NCT04595565. Registry record. Accessed 8 September 2026. https://clinicaltrials.gov/study/NCT04595565

[30] Schmid P, et al. TROPION-Breast05: a randomized phase III study of Dato-DXd with or without durvalumab versus chemotherapy plus pembrolizumab in patients with PD-L1-high locally recurrent inoperable or metastatic triple-negative breast cancer. Ther Adv Med Oncol. 2025;17:17588359251327992. https://pubmed.ncbi.nlm.nih.gov/40297626/

[31] Modi S, et al. Trastuzumab Deruxtecan in Previously Treated HER2-Low Advanced Breast Cancer. N Engl J Med. 2022;387:9–20. PMID 35665782. https://pubmed.ncbi.nlm.nih.gov/35665782/

[32] ClinicalTrials.gov. DS8201a and Pembrolizumab in Participants With Locally Advanced/Metastatic Breast or Non-Small Cell Lung Cancer. NCT04042701. Registry record. Accessed 8 September 2026. https://clinicaltrials.gov/study/NCT04042701

[33] Trastuzumab deruxtecan With Pembrolizumab in Previously Treated HER2-Expressing Advanced or Metastatic Breast Cancer: Interim Analyses of the Breast Cohorts from the Open-Label, Multicenter, Phase 1b Study DS8201-A-U106. Clin Cancer Res. 2025;31(12 Suppl):P5-07-29. Conference abstract. https://aacrjournals.org/clincancerres/article-split/31/12_Supplement/P5-07-29/752921/Abstract-P5-07-29-Trastuzumab-deruxtecan-With

[34] Schmid P, et al. First-line durvalumab in combination with trastuzumab deruxtecan in women with locally advanced unresectable or metastatic, hormone-receptor-negative, HER2-low breast cancer: multicenter, open-label, phase 1b/2 BEGONIA platform trial. Nat Cancer. 2026. doi:10.1038/s43018-026-01181-8. https://pubmed.ncbi.nlm.nih.gov/42259988/

[102] ClinicalTrials.gov. A Study Evaluating the Efficacy and Safety of Multiple Treatment Combinations in Patients With Metastatic or Locally Advanced Breast Cancer (Morpheus-panBC). NCT03424005. Registry record. Accessed 8 September 2026. https://clinicaltrials.gov/study/NCT03424005

[103] Bardia A, et al. Sacituzumab Govitecan in Metastatic Triple-Negative Breast Cancer. N Engl J Med. 2021;384:1529–1541. https://doi.org/10.1056/NEJMoa2028485

[104] Schmid P, et al. Datopotamab deruxtecan (Dato-DXd) in combination with durvalumab as first-line treatment for unresectable locally advanced or metastatic triple-negative breast cancer: results from arms 7 and 8 of the phase Ib/II BEGONIA study. Ann Oncol. 2026;37:1081–1092. https://doi.org/10.1016/j.annonc.2026.05.693
