# Supplementary material for: Precision immuno-oncology in triple-negative breast cancer: from PD-L1 to multimodal biomarkers, ADC–ICI combinations, and global real-world integration
Source: Front Immunol. 2026 Oct 12;17:1911879. doi: 10.3389/fimmu.2026.1911879 (PMC13696950; doi:10.3389/fimmu.2026.1911879)
Supplement: Supplementary file 2 [file Table2.docx]

Supplementary Table 2 Biomarker evidence and permissible interpretation

| **Biomarker and setting** | **Measurement** | **Supported role** | **Limit on treatment action** | **Implementation and validation** | **Refs.** |
| --- | --- | --- | --- | --- | --- |
| PD-L1 for advanced TNBC pembrolizumab combinations | Validated CPS assay; clinically relevant threshold ≥10 | Regimen-specific selection linked to randomized evidence and labeling | Not the early-stage KEYNOTE-522 eligibility threshold | Assay and scoring quality; tissue adequacy; specimen timing; access and turnaround. | [2, 23] |
| PD-L1 for atezolizumab historical TNBC evidence | SP142 immune-cell area score ≥1% | Identifies the population used in IMpassion130 | Not CPS; assays are noninterchangeable; United States indication withdrawn; local status matters | Different scoring denominators; platform availability; local regulatory status. | [15, 16, 18] |
| Stromal TILs | Proportion of tumor stromal area occupied by lymphocytes | Prognostic association in early TNBC, including patients without systemic chemotherapy | Does not establish incremental ICI benefit or a safe omission threshold | Sampling, reader variability and harmonized stromal scoring. | [38] |
| Spatial immune features | Multiplex cellular measurements and spatial relationships | Trial-associated TNBC response analyses | Research only; lock assay/model, validate externally and prospectively test the treatment decision. | Tissue selection, segmentation and pipeline variability; cost and limited center access. | [3] |
| TLS and B-cell features | Structure, maturity and location; tissue or expression measurements | Mechanistic and response associations in other tumor types | Indirect evidence; no universal TNBC treatment-selection cutoff | Definition, maturity and location; sampling variability. | [39] |
| Germline BRCA1/2 versus HRD scar | Pathogenic variants versus broader genomic scar assays | Specific BRCA-defined uses of PARP inhibitors; distinct from ICI prediction | Do not equate HRD with BRCA mutation or assume added ICI benefit | Variant interpretation, counseling and access; genomic scars need not reflect current repair function. | [44, 45, 46] |
| TMB-high / MSI-high | Authorized molecular assays | Tissue-agnostic diagnostic eligibility under applicable labeling | Check full clinical indication; not a general first-line substitute for PD-L1 in TNBC | Assay validation, tissue adequacy and full indication-specific eligibility. | [47, 48] |
| ctDNA residual disease / kinetics | Serial blood testing with specified assay and timing | Prognostic molecular-risk information; c-TRAK TN evaluated intervention feasibility, not safe treatment omission. | No prospective interventional evidence supports omitting adjuvant pembrolizumab solely because ctDNA is negative. Positivity alone does not identify an effective escalation. | Assay heterogeneity; low shedding and false negatives; clonal hematopoiesis; serial sampling, turnaround and cost. | [8, 9, 51, 53–55] |
| Immune transcriptomic or AI composite model | Prespecified gene set or multimodal model | Candidate research prediction; incremental value must be tested against clinicopathological variables plus setting-appropriate PD-L1. | Retrospective discrimination is insufficient. Require calibration, independent validation and prospective utility; treatment prediction needs a comparator and interaction analysis. | Feature selection, imputation and tuning within training; separate patients across datasets; evaluate missing modalities and a simpler fallback model. | [3, 49, 50, 60] |
| Gut microbiome | Sequencing with medication and specimen controls | Interventional signal in resistant melanoma | Indirect evidence, not validated TNBC selection or routine FMT indication | Diet, medication, geography, handling and contamination. | [57] |
| CAR target-antigen density | Construct- and assay-specific flow cytometry | CD70 abundance related to AML CAR-T activity in Cheng et al. | AML, not TNBC; MFI ratio around 1.6 cannot be transferred to PD-L1 or other CAR products | Construct, assay and normal-tissue context; no portable threshold. | [68] |
| Trop-2 / ADC target abundance | Target expression in a defined specimen and assay | Exploratory ASCENT biomarker analyses; resistance observations | No universal exclusion threshold; target delivery and payload sensitivity are distinct | Archival sampling, heterogeneity, delivery and payload resistance. | [35, 36] |

Prognostic association is not equivalent to prediction of differential treatment benefit. PD-L1 is not required to select patients for the KEYNOTE-522 regimen; metastatic thresholds must not be imported into that setting. No prospective interventional evidence supports omitting its adjuvant pembrolizumab component solely because ctDNA is negative.

Missing or technically failed tests must not be classified as negative. Serial ctDNA analyses restricted to complete sampling may select healthier or better-resourced patients. Report eligible, sampled and successfully tested denominators at each time point, reasons for missingness and sensitivity analyses for informative missingness. There is no single harmonized routine TNBC MRD sampling schedule. [9, 51, 54, 55]

Advanced spatial, multi-omics and microbiome measurements, and ctDNA-MRD-guided ICI escalation or omission, remain research applications. Require incremental value beyond a clinicopathological baseline with setting-appropriate PD-L1, independent validation and prospective clinical utility. Assess assay access, cost, turnaround and the proportion receiving a usable result.

Abbreviations: TILs, tumor-infiltrating lymphocytes; TLS, tertiary lymphoid structures; HRD, homologous recombination deficiency; TMB, tumor mutational burden; MSI, microsatellite instability; ctDNA, circulating tumor DNA; MRD, molecular residual disease; FMT, fecal microbiota transplantation; AML, acute myeloid leukemia; MFI, mean fluorescence intensity; AI, artificial intelligence; CAR, chimeric antigen receptor; CPS, combined positive score; ICI, immune checkpoint inhibitor; ADC, antibody–drug conjugate; TNBC, triple-negative breast cancer.

# References for this table

[2] Cortés J, et al. Pembrolizumab plus Chemotherapy in Advanced Triple-Negative Breast Cancer. N Engl J Med. 2022;387:217–226. https://doi.org/10.1056/NEJMoa2202809

[3] Wang XQ, et al. Spatial predictors of immunotherapy response in triple-negative breast cancer. Nature. 2023;621:868–876. PMID 37674077. https://pubmed.ncbi.nlm.nih.gov/37674077/

[8] Schmid P, et al. Pembrolizumab for Early Triple-Negative Breast Cancer. N Engl J Med. 2020;382:810–821. https://doi.org/10.1056/NEJMoa1910549

[9] Turner NC, et al. Results of the c-TRAK TN trial: a clinical trial utilising ctDNA mutation tracking to detect molecular residual disease and trigger intervention in patients with moderate- and high-risk early-stage triple-negative breast cancer. Ann Oncol. 2023. PMID 36423745. https://pubmed.ncbi.nlm.nih.gov/36423745/

[15] Schmid P, et al. Atezolizumab and Nab-Paclitaxel in Advanced Triple-Negative Breast Cancer. N Engl J Med. 2018;379:2108–2121. https://doi.org/10.1056/NEJMoa1809615

[16] Rugo HS, et al. PD-L1 Immunohistochemistry Assay Comparison in Atezolizumab Plus nab-Paclitaxel-Treated Advanced Triple-Negative Breast Cancer. J Natl Cancer Inst. 2021. PMID 34097070. https://pubmed.ncbi.nlm.nih.gov/34097070/

[18] US Food and Drug Administration. CDER accelerated approval records including withdrawn indications. Regulatory record. Accessed 8 September 2026. https://www.fda.gov/media/151146/download

[23] US Food and Drug Administration. FDA approves sacituzumab govitecan-hziy as monotherapy and in combination with pembrolizumab for first-line treatment of triple-negative breast cancer. 24 June 2026. Accessed 8 September 2026. https://www.fda.gov/drugs/resources-information-approved-drugs/fda-approves-sacituzumab-govitecan-hziy-monotherapy-and-combination-pembrolizumab-first-line

[35] Bardia A, et al. Biomarker analyses in the phase III ASCENT study of sacituzumab govitecan versus chemotherapy in patients with metastatic triple-negative breast cancer. Ann Oncol. 2021. PMID 34116144. https://pubmed.ncbi.nlm.nih.gov/34116144/

[36] Coates JT, et al. Parallel Genomic Alterations of Antigen and Payload Targets Mediate Polyclonal Acquired Clinical Resistance to Sacituzumab Govitecan in Triple-Negative Breast Cancer. Cancer Discov. 2021;11:2436–2445. https://pubmed.ncbi.nlm.nih.gov/34404686/

[38] Leon-Ferre RA, et al. Tumor-Infiltrating Lymphocytes in Triple-Negative Breast Cancer. JAMA. 2024;331:1135–1144. https://doi.org/10.1001/jama.2024.3056

[39] Helmink BA, et al. B cells and tertiary lymphoid structures promote immunotherapy response. Nature. 2020. https://doi.org/10.1038/s41586-019-1922-8

[44] Robson M, et al. Olaparib for Metastatic Breast Cancer in Patients with a Germline BRCA Mutation. N Engl J Med. 2017. PMID 28792849. https://pubmed.ncbi.nlm.nih.gov/28792849/

[45] Tutt ANJ, et al. Adjuvant Olaparib for Patients with BRCA1- or BRCA2-Mutated Breast Cancer. N Engl J Med. 2021. PMID 34081848. https://pubmed.ncbi.nlm.nih.gov/34081848/

[46] Tan TJ, et al. Phase II DORA Study of Olaparib with or without Durvalumab as a Chemotherapy-Free Maintenance Strategy in Platinum-Pretreated Advanced Triple-Negative Breast Cancer. Clin Cancer Res. 2024;30:1240–1247. doi:10.1158/1078-0432.CCR-23-2513. https://pubmed.ncbi.nlm.nih.gov/38236575/

[47] US Food and Drug Administration. FDA approves pembrolizumab for adults and children with TMB-H solid tumors. 16 June 2020. Accessed 8 September 2026. https://www.fda.gov/drugs/drug-approvals-and-databases/fda-approves-pembrolizumab-adults-and-children-tmb-h-solid-tumors

[48] US Food and Drug Administration. List of FDA-Authorized Companion Diagnostic Devices (In Vitro and Imaging Tools). Accessed 8 September 2026. https://www.fda.gov/medical-devices/in-vitro-diagnostics/list-fda-authorized-companion-diagnostic-devices-in-vitro-and-imaging-tools

[49] Simon RM, et al. Use of archived specimens in evaluation of prognostic and predictive biomarkers. J Natl Cancer Inst. 2009;101:1446–1452. https://doi.org/10.1093/jnci/djp335

[50] Simon R, et al. Pitfalls in the use of DNA microarray data for diagnostic and prognostic classification. J Natl Cancer Inst. 2003;95:14–18. https://doi.org/10.1093/jnci/95.1.14

[51] Pascual J, et al. ESMO recommendations on the use of circulating tumour DNA assays for patients with cancer: a report from the ESMO Precision Medicine Working Group. Ann Oncol. 2022;33:750–768. https://doi.org/10.1016/j.annonc.2022.05.520

[53] Radovich M, et al. Association of circulating tumor DNA and circulating tumor cells after neoadjuvant chemotherapy with disease recurrence in patients with triple-negative breast cancer. JAMA Oncol. 2020;6:1410–1415. https://doi.org/10.1001/jamaoncol.2020.2295

[54] Shaw JA, et al. Serial postoperative circulating tumor DNA assessment has strong prognostic value during long-term follow-up in patients with breast cancer. JCO Precis Oncol. 2024;8:e2300456. https://doi.org/10.1200/PO.23.00456

[55] Hu Y, et al. False-positive plasma genotyping due to clonal hematopoiesis. Clin Cancer Res. 2018;24:4437–4443. https://doi.org/10.1158/1078-0432.CCR-18-0143

[57] Davar D, et al. Fecal microbiota transplant overcomes resistance to anti-PD-1 therapy in melanoma patients. Science. 2021;371:595–602. PMID 33542131. https://pubmed.ncbi.nlm.nih.gov/33542131/

[60] Collins GS, et al. TRIPOD+AI statement: updated guidance for reporting clinical prediction models that use regression or machine learning methods. BMJ. 2024;385:e078378. https://doi.org/10.1136/bmj-2023-078378

[68] Cheng J, et al. Preclinical development and evaluation of nanobody-based CD70-specific CAR T cells for the treatment of acute myeloid leukemia. Cancer Immunol Immunother. 2023;72:2331–2346. https://doi.org/10.1007/s00262-023-03422-6
