# Supplementary material for: Precision immuno-oncology in triple-negative breast cancer: from PD-L1 to multimodal biomarkers, ADC–ICI combinations, and global real-world integration
Source: Front Immunol. 2026 Oct 12;17:1911879. doi: 10.3389/fimmu.2026.1911879 (PMC13696950; doi:10.3389/fimmu.2026.1911879)
Supplement: Supplementary file 3 [file Table3.docx]

Supplementary Table 3 Resistance directed hypotheses and clinical evidence

| **Biological hypothesis** | **Intervention and measurement** | **Human evidence and model context** | **Limits and next research requirement** | **Refs.** |
| --- | --- | --- | --- | --- |
| WNT-associated immune exclusion | Investigate pathway activity with spatial immune infiltration | Pan-cancer association with a noninflamed state | No validated TNBC WNT-based prescribing rule or demonstrated routine rescue | [62] |
| PI3K-AKT pathway alteration | AKT-directed treatment; characterize alteration and functional context | IPATunity130 randomized phase III: no PFS benefit from adding ipatasertib to paclitaxel in alteration-selected advanced TNBC. | The comparison did not include an ICI. Not a test of ICI-resistance reversal; a pathway alteration alone does not validate an AKT–ICI regimen. | [70] |
| B2M / JAK pathway loss | Characterize functional loss versus reversible low expression | Paired acquired-resistance observations in melanoma | Candidate mechanisms for TNBC: prevalence, allelic/functional impact and treatment relevance require TNBC-specific validation. Epigenetic treatment cannot be assumed to repair structural B2M loss. | [63] |
| Reversible epigenetic suppression | HDAC/DNMT modulation with paired expression assessment | Phase Ib entinostat + nivolumab + ipilimumab in 24 women with HER2-negative breast cancer, including 12 with TNBC; four responses among ten evaluable TNBC participants. | Small uncontrolled combination; entinostat contribution not isolated. Establish a reversible baseline defect, pharmacodynamic correction and benefit with an appropriate comparator. | [67] |
| Insufficient CAR target abundance | Decitabine/chidamide; CD70 density and CAR function | Preclinical AML evidence; explicitly indirect | No TNBC HLA restoration or transferable MFI threshold demonstrated | [68] |
| Adenosine-mediated suppression | Oleclumab; CD73-related measurements | Breast cancer model rationale; randomized phase II SYNERGY: primary endpoint not met (24-week clinical benefit 43% versus 44%). | Does not support routine oleclumab addition to the tested regimen. Future studies need functional selection and justification of a different drug, schedule or population. | [69, 71, 72] |
| DNA damage-driven immune priming | PARP inhibition; STING pathway and immune recruitment | BRCA-deficient TNBC models support STING-related recruitment. TOPACIO: single-arm TNBC; MEDIOLA: broader BRCA-mutated HER2-negative population; DORA: randomized, noncomparative phase II after platinum benefit, with broader receptor criteria than strict TNBC. | Added ICI benefit is unestablished. Separate direct PARP activity from immune effects; use a PARP-based comparator and account for prior platinum/PARP exposure. | [46, 75–77] |
| Insufficient local priming | Short induction or radiotherapy followed by/with ICI | TONIC: randomized but noncomparative phase II in metastatic TNBC; separate small pembrolizumab–RT phase II study. | The optimal regimen, reproducible abscopal effects and survival benefit remain unestablished; comparative studies with prespecified local and systemic endpoints are needed. | [78, 79] |
| Local antigen release by oncolytic virus | T-VEC with chemotherapy or atezolizumab; injectable disease | Phase II T-VEC + neoadjuvant chemotherapy in TNBC did not test an ICI. PROMETEO: single-arm T-VEC + atezolizumab in mixed residual HER2-negative breast cancer. | Regimens are distinct; comparative benefit and immune contribution remain unproven. Separate injected from noninjected lesion outcomes and test systemic effects. | [80, 81] |
| Complementary checkpoint inhibition | Nivolumab ± ipilimumab; immune activation | BELLINI nonrandomized phase II in early TNBC | Not proof of routine chemotherapy omission or efficacy of all alternative checkpoint classes | [82] |
| Insufficient antigen-specific priming | Individualized neoantigen DNA or mRNA vaccination; measure specific T-cell responses | Early TNBC studies; DNA vaccine n=18 and mRNA vaccine n=14 | Immunogenicity does not establish recurrence prevention or reversal of exclusion | [85, 86] |

These entries are research hypotheses, not a treatment algorithm. Mechanistic rationale, pharmacodynamic change or response in an uncontrolled study does not establish reversal of ICI resistance. Non-TNBC evidence is labeled explicitly.

Negative randomized findings apply to the tested intervention, comparator and population. SYNERGY did not meet its phase II primary endpoint; IPATunity130 did not demonstrate a phase III PFS benefit in the cited comparison, which contained no ICI. Neither result should be generalized to every intervention affecting the same pathway.

Abbreviations: HDAC, histone deacetylase; DNMT, DNA methyltransferase; A2AR, adenosine A2A receptor; STING, stimulator of interferon genes; RT, radiotherapy; AML, acute myeloid leukemia; CAR, chimeric antigen receptor; MFI, mean fluorescence intensity; ICI, immune checkpoint inhibitor; PFS, progression-free survival; TNBC, triple-negative breast cancer; T-VEC, talimogene laherparepvec.

# References for this table

[46] Tan TJ, et al. Phase II DORA Study of Olaparib with or without Durvalumab as a Chemotherapy-Free Maintenance Strategy in Platinum-Pretreated Advanced Triple-Negative Breast Cancer. Clin Cancer Res. 2024;30:1240–1247. doi:10.1158/1078-0432.CCR-23-2513. https://pubmed.ncbi.nlm.nih.gov/38236575/

[62] Luke JJ, et al. WNT/β-catenin Pathway Activation Correlates with Immune Exclusion across Human Cancers. Clin Cancer Res. 2019. PMID 30635339. https://pubmed.ncbi.nlm.nih.gov/30635339/

[63] Zaretsky JM, et al. Mutations Associated with Acquired Resistance to PD-1 Blockade in Melanoma. N Engl J Med. 2016;375:819–829. https://doi.org/10.1056/NEJMoa1604958

[67] Roussos Torres ET, et al. Entinostat, nivolumab and ipilimumab for women with advanced HER2-negative breast cancer: a phase Ib trial. Nat Cancer. 2024. PMID 38355777. https://pubmed.ncbi.nlm.nih.gov/38355777/

[68] Cheng J, et al. Preclinical development and evaluation of nanobody-based CD70-specific CAR T cells for the treatment of acute myeloid leukemia. Cancer Immunol Immunother. 2023;72:2331–2346. https://doi.org/10.1007/s00262-023-03422-6

[69] Buisseret L, et al. Paclitaxel plus carboplatin and durvalumab with or without oleclumab for women with previously untreated locally advanced or metastatic triple-negative breast cancer: the randomized SYNERGY phase I/II trial. Nat Commun. 2023;14:7018. https://doi.org/10.1038/s41467-023-42744-y

[70] Dent RA, et al. Ipatasertib plus Paclitaxel for Patients with PIK3CA/AKT1/PTEN-Altered Locally Advanced Unresectable or Metastatic Triple-Negative Breast Cancer in the IPATunity130 Phase III Trial. Clin Cancer Res. 2024;30:4329–4338. doi:10.1158/1078-0432.CCR-24-0465. https://pubmed.ncbi.nlm.nih.gov/39058425/

[71] Stagg J, et al. Anti-CD73 antibody therapy inhibits breast tumor growth and metastasis. Proc Natl Acad Sci USA. 2010;107:1547–1552. https://doi.org/10.1073/pnas.0908801107

[72] Loi S, et al. CD73 promotes anthracycline resistance and poor prognosis in triple negative breast cancer. Proc Natl Acad Sci USA. 2013;110:11091–11096. https://doi.org/10.1073/pnas.1222251110

[75] Pantelidou C, et al. PARP Inhibitor Efficacy Depends on CD8+ T-cell Recruitment via Intratumoral STING Pathway Activation in BRCA-Deficient Models of Triple-Negative Breast Cancer. Cancer Discov. 2019. PMID 31015319. https://pubmed.ncbi.nlm.nih.gov/31015319/

[76] Vinayak S, et al. Open-label clinical trial of niraparib combined with pembrolizumab for treatment of advanced or metastatic triple-negative breast cancer. JAMA Oncol. 2019;5:1132–1140. https://doi.org/10.1001/jamaoncol.2019.1029

[77] Domchek SM, et al. Olaparib and durvalumab in patients with germline BRCA-mutated metastatic breast cancer (MEDIOLA). Lancet Oncol. 2020;21:1155–1164. https://doi.org/10.1016/S1470-2045(20)30324-7

[78] Voorwerk L, et al. Immune induction strategies in metastatic triple-negative breast cancer to enhance the sensitivity to PD-1 blockade: the TONIC trial. Nat Med. 2019. PMID 31086347. https://pubmed.ncbi.nlm.nih.gov/31086347/

[79] Ho AY, et al. A phase 2 clinical trial assessing the efficacy and safety of pembrolizumab and radiotherapy in patients with metastatic triple-negative breast cancer. Cancer. 2020. PMID 31747077. https://pubmed.ncbi.nlm.nih.gov/31747077/

[80] Soliman HH, et al. Oncolytic T-VEC virotherapy plus neoadjuvant chemotherapy in nonmetastatic triple-negative breast cancer: a phase 2 trial. Nat Med. 2023. doi:10.1038/s41591-023-02210-0. https://pubmed.ncbi.nlm.nih.gov/36759673/

[81] Pascual T, et al. Talimogene laherparepvec and atezolizumab in HER2-negative breast cancer following neoadjuvant chemotherapy: a window-of-opportunity phase II trial (SOLTI-1503 PROMETEO). Nat Commun. 2026;17:2817. doi:10.1038/s41467-026-69222-5. https://pubmed.ncbi.nlm.nih.gov/41698899/

[82] Nederlof I, et al. Neoadjuvant nivolumab or nivolumab plus ipilimumab in early-stage triple-negative breast cancer: a phase 2 adaptive trial. Nat Med. 2024. https://doi.org/10.1038/s41591-024-03249-3

[85] Zhang X, et al. Neoantigen DNA vaccines are safe, feasible, and induce neoantigen-specific immune responses in triple-negative breast cancer patients. Genome Med. 2024;16:131. https://pubmed.ncbi.nlm.nih.gov/39538331/

[86] Sahin U, et al. Individualized mRNA vaccines evoke durable T cell immunity in adjuvant TNBC. Nature. 2026;651:1088–1096. https://doi.org/10.1038/s41586-025-10004-2
